# Connectivity-based parcellation increases network detection sensitivity in resting state fMRI: An investigation into the cingulate cortex in autism

**DOI:** 10.1016/j.nicl.2016.03.016

**Published:** 2016-03-25

**Authors:** Joshua H. Balsters, Dante Mantini, Matthew A.J. Apps, Simon B. Eickhoff, Nicole Wenderoth

**Affiliations:** a Neural Control of Movement Laboratory, Department of Health Sciences and Technology, ETH Zurich, Switzerland; b Department of Experimental Psychology, University of Oxford, Oxford OX1 3UD, UK; c Movement Control and Neuroplasticity Research Group, Department of Kinesiology, KU Leuven, Belgium; d Institute of Neuroscience and Medicine (INM-1), Research Center Jülich, Germany; e Institute of Clinical Neuroscience and Medical Psychology, Heinrich-Heine University Düsseldorf, Germany

**Keywords:** Autism Spectrum Disorder, Cingulate cortex, Resting state fMRI, Meta-Analytic Connectivity Modeling

## Abstract

Although resting state fMRI (RS-fMRI) is increasingly used to generate biomarkers of psychiatric illnesses, analytical choices such as seed size and placement can lead to variable findings. Seed placement especially impacts on RS-fMRI studies of Autism Spectrum Disorder (ASD), because individuals with ASD are known to possess more variable network topographies. Here, we present a novel pipeline for analysing RS-fMRI in ASD using the cingulate cortex as an exemplar anatomical region of interest. Rather than using seeds based on previous literature, or gross morphology, we used a combination of structural information, task-independent (RS-fMRI) and task-dependent functional connectivity (Meta-Analytic Connectivity Modeling) to partition the cingulate cortex into six subregions with unique connectivity fingerprints and diverse behavioural profiles. This parcellation was consistent between groups and highly replicable across individuals (up to 93% detection) suggesting that the organisation of cortico-cingulo connections is highly similar between groups. However, our results showed an age-related increase in connectivity between the anterior middle cingulate cortex and right lateral prefrontal cortex in ASD, whilst this connectivity decreased in controls. There was also a Group × Grey Matter (GM) interaction, showing increased connectivity between the anterior cingulate cortex and the rectal gyrus in concert with increasing rectal gyrus GM in controls. By comparing our approach to previously established methods we revealed that our approach improves network detection in both groups, and that the ability to detect group differences using 4 mm radius spheres varies greatly with seed placement. Using our multi-modal approach we find disrupted cortico-cingulo circuits that, based on task-dependent information, may contribute to ASD deficits in attention and social interaction. Moreover, we highlight how more sensitive approaches to RS-fMRI are crucial for establishing robust and reproducible connectivity-based biomarkers in psychiatric disorders.

## Highlights

• Cingulate cortex connectivity is often implicated as a marker of Autism (ASD).
• However, these differences may be due to analytical choices such as seed placement.
• Our new multi-modal pipeline for resting state fMRI improved network detection in both groups.
• Cingulate parcellation was highly replicable across individuals (up to 93% detection).
• We show developmental trajectories of cortico-cingulo connectivity that may underlie attention and social deficits in ASD.

## 1 Introduction

Spontaneous, or resting state (RS), fluctuations in the blood oxygenation level-dependent (BOLD) signal are increasingly used as a tool to investigate brain connectivity and generate biomarkers of psychiatric disorders (Alaerts et al., 2014, Di Martino et al., 2014, Kelly et al., 2012). However, the unconstrained nature of spontaneous fluctuations means that they are susceptible to non-neural factors such as head movement, respiration, and MRI artifacts, leading to a moderate test-retest reliability (Shehzad et al., 2009, Thomason et al., 2011, Zuo et al., 2010) and reducing the utility of RS-fMRI as a diagnostic tool. For example, evidence from RS-fMRI provided great support for the developmental disconnection hypothesis (Courchesne and Pierce, 2005), which posited that individuals with ASD show decreased long-range neural connectivity in parallel with increased short-range connectivity. However, there is now some debate about whether these differences in connectivity are actually due to differences in small head movements (Deen and Pelphrey, 2012, Koldewyn et al., 2014, Tyszka et al., 2014).

There has been an increasing effort to quantify the impact of various pre-processing steps and provide recommendations (Bright and Murphy, 2015, Power et al., 2012, Shirer et al., 2015, Yan et al., 2013), however, one issue that has not been discussed as widely is the generation of regions of interest (seeds) in RS-fMRI. Smith et al. (2011) showed that the incorrect selection of a seed region had the greatest potential to reduce network detection. Specifically, it was demonstrated that when a seed crossed a functional boundary by as little as 20% the sensitivity of detecting connections dropped dramatically (< 20% detection sensitivity regardless of the method). Given that Hahamy et al. (2015) recently demonstrated that individuals with ASD possess more variable spatial representations of resting state networks, we suggest that some RS-fMRI differences in ASD may not be due to reduced connectivity but variability in spatial representations of networks which traditional seed-based approaches are not sensitive enough to detect.

RS-fMRI is increasingly being used as a tool to partition gross morphological regions into functional sub-units based on common temporal dynamics (Eickhoff et al., 2015, Gordon et al., 2014, Wig et al., 2014, Yeo et al., 2011). This data-driven approach allows one to generate more accurate seed regions with common connectivity fingerprints. This is especially useful for anatomical regions that are functionally and cytoarchitectonically heterogeneous, but do not possess gross morphological landmarks to delineate these boundaries, such as the cingulate cortex. The cingulate cortex has been implicated in a wide range of behaviours including motor control (Amiez and Petrides, 2012, Paus, 2001), cognitive control (Shenhav et al., 2013), conflict monitoring (Botvinick et al., 1999, Botvinick, 2007), economic decision making (Kolling et al., 2014, Rushworth and Behrens, 2008), and social cognition (Apps et al., 2015, Apps et al., 2013, Behrens et al., 2009, Chang et al., 2013, Lockwood et al., 2015). This functional variability is a likely consequence of the variable cytoarchitecture and diverse connectivity fingerprints present in the cingulate cortex. Although it is not currently possible to detect cytoarchitectonic boundaries within the cingulate cortex using fMRI alone, it is possible to partition the cingulate cortex into sub-regions with unique connectivity fingerprints (Beckmann et al., 2009, Neubert et al., 2015). This makes the cingulate cortex an excellent model to establish our data-driven parcellation approach.

As well as being an excellent model for investigating connectivity-based parcellation, the cingulate cortex is also one of the most reported structures implicated in ASD (Cauda et al., 2011). There is a large body of literature highlighting that individuals with ASD have differences in cingulate anatomy and cytoarchitecture, cingulate connectivity as measured by RS-fMRI and DTI, and cingulate function. Post mortem studies of the anterior cingulate cortex (ACC) have shown that Area 32 in individuals with ASD has decreased long range fibres but increased short-range connections (Zikopoulos and Barbas, 2010), whilst Area 24b showed significantly decreased neuron size and area in ASD, and Area 24c shows decreased neuron density in layer V—VI in ASD (Simms et al., 2009). Simms et al. (2009) also investigated the presence of Von Economo Neurons (VENs), and found that three of the nine cases presented with a significant increase in VENs whilst the remaining six presented with a significant decrease in VENs. The heterogeneity present in these post-mortem analyses is also present in-vivo structural analyses such as voxel based morphology (VBM), as well as cortical thickness and surface area. In a meta-analysis of VBM studies by Cauda et al. (2011) they found that individuals with ASD showed a significant increase in subgenual ACC grey matter volume (putatively Area 33). However, two other meta-analyses conducted by Nickl-Jockschat et al. (2012) and Via et al. (2011) did not find any differences in cingulate cortex grey matter (GM) volume in ASD. It is possible that this variability is due to differences in the developmental trajectory of individuals with ASD, and depending on the age of acquisition you may or may not find group differences (Uddin et al., 2013). Indeed, Duerden et al. (2011) found that children and adolescents with ASD were more likely to show a difference in cortical thickness in the ACC compared to adults with ASD. Greimel et al. (2013) also showed different developmental trajectories in GM volume in the middle cingulate cortex (MCC), although similar studies by Foster et al. (2015) and Zielinski et al. (2014) did not find Group × Age interactions in the cingulate cortex.

There is also a large body of literature using fMRI and functional connectivity (RS-fMRI) to investigate the cingulate cortex in ASD. However, the majority of these studies have investigated the default mode network (DMN) in ASD rather than the cingulate cortex specifically. Like the cingulate cortex, the DMN has been linked to social cognition and theory of mind processes (Buckner et al., 2008), making the DMN a target for investigation in ASD. Kennedy and Courchesne (2008a) had individuals with ASD make true/false statements about themselves or someone else, and these statements were either about personality traits (internal) or objects (external). They found a task-independent reduction of activity in the ACC in ASD (bordering the subgenual ACC (sACC) and pregenual (pACC)). Group differences in internal vs external task judgments were found in the superior medial prefrontal cortex (putatively BA 9) and dorsal posterior cingulate cortex (dPCC). All of these group differences (both task-dependent and task-independent) fell within the DMN. A meta-analysis of studies using both social and non-social paradigms in ASD also found differences within cingulate nodes of the DMN (Di Martino et al., 2009). Specifically, pACC and ventral PCC (vPCC) were more active in controls compared to ASD individuals during social paradigms. RS-fMRI studies investigating the DMN in ASD typically found reduced connectivity between the anterior and posterior cingulate nodes (Assaf et al., 2010, Di Martino et al., 2013, Kennedy and Courchesne, 2008b, Kennedy et al., 2006, Starck et al., 2013, Washington et al., 2014). However, as with the previously mentioned structural studies, this finding has not always been replicated (Lynch et al., 2013, Monk et al., 2009, Tyszka et al., 2014). As mentioned previously, it is possible that the age of the sample may influence the ability to detect group differences in RS-fMRI as well as structural imaging (Uddin et al., 2013). Several recent investigations of functional connectivity in ASD have suggested that careful characterization of participant age is necessary in order to avoid obscuring developmental group differences (Abbott et al., 2015, Nomi and Uddin, 2015, Uddin et al., 2013). Given that differences in age appear to have such a strong impact on structural and functional analyses of individuals with ASD, we have taken steps to match groups for age and include age as a variable of interest in order to investigate divergent developmental trajectories.

Here, we provide a novel multi-modal pipeline for investigating anatomical variability in clinical populations. Specifically, we used a large dataset of RS-fMRI data from individuals with ASD and matched controls (*N* = 260, 50% ASD) to investigate the parcellation and connectivity of the cingulate cortex. First, we hypothesise that seeds generated using connectivity-based parcellation will improve network detection and increase within network connectivity strength compared to traditional methods. We also hypothesise that these seeds will have distinct behavioural profiles established using meta-analytic tools. Finally, this approach allowed us to address whether: (i) group differences exist in the organization of the cingulate cortex (i.e. presence or absence of cingulate subregions in a clinical population), or (ii) whether the architecture of connections is the same between groups but connectivity strength differs between groups.

## 2 Methods

**Fig. 1:**
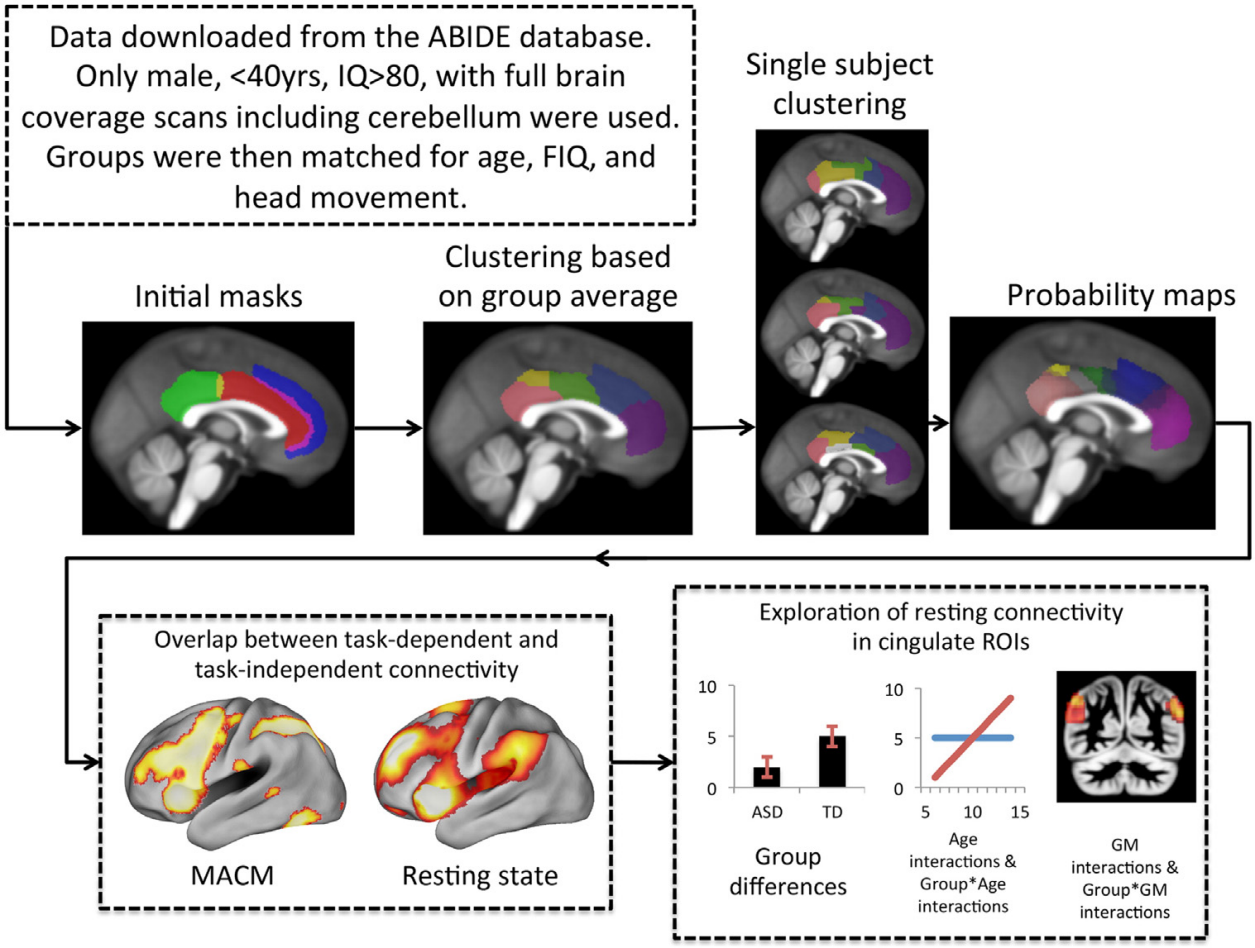
Overview of the analysis pipeline. Data were extracted from the ABIDE database (Di Martino et al., 2013) and cortical masks were extracted from the Harvard-Oxford cortical atlas. For each subject connectivity matrices were calculated showing connectivity between the cortical mask (cingulate cortex) and all grey matter voxels. These matrices were averaged and a hierarchical clustering algorithm was used to cluster voxels with similar connectivity patterns. This group average result was then forced onto single subject connectivity matrices and dice similarity was used establish how replicable the group template was for each individual subject. Individual subject clustering solutions were binarised and summed together to create probability maps. These probability maps were used for all subsequent analyses.

Fig. 1 provides an overview of the analytical steps. Individual RS-fMRI sessions were downloaded from the ABIDE database in order to create a large, matched cohort. For each individual, we created a connectivity matrix establishing the connectivity strength between voxels within the mask (i.e., cingulate cortex) and all GM voxels in the brain including the original mask. We then used a hierarchical clustering approach to partition the cingulate cortex into sub-regions with unique connectivity patterns based on the group average of the previously generated connectivity matrices. This group-based solution was then back-projected on individual subjects in order to investigate replicability of the group solution at the individual level (i.e. how often does each cluster in the group solution present itself in an individual subject). It was also possible at this stage to generate probability maps of cingulate parcellations in order to quantify variability in the size and shape of each parcellation across individuals. Parcellations based on unique connectivity fingerprints should also have unique functional profiles. We used the Brainmap database to establish functional profiles for each cingulate parcellation. We also used Meta-Analytic Connectivity Modeling (MACM) to generate task-dependent connectivity maps to compare with task-independent connectivity maps. The aim of these previous steps was to establish the reliability of the cingulate parcellation solution. Finally, we used non-parametric permutation testing to determine whether connectivity strength within each cingulate parcellation's connectivity fingerprint was significantly altered in ASD, including Group × Age and Group × GM interactions.

### 2.1 Participants

Data were extracted from the ABIDE database (Di Martino et al., 2013) using the same exclusion criteria as Di Martino et al. (2013). Subjects were excluded if they were: female, > 40 years old, IQ < 80, or moved excessively (mean framewise displacement (FD) > 0.5 mm). It was important to remove these individuals as they were not well represented in the ABIDE database and their inclusion in the analyses would introduce additional variability or a potential bias. Given our interest in developmental trajectories we excluded a small number of older individuals (> 40 years old) who all came from one centre and might artificially drive age effects or Group × Age interactions. We additionally removed subjects with poor GM segmentation as this would impact on quality of the DARTEL template and subsequent normalisation. GM segmentation was assessed using the squared distance of each image to the sample mean (tool available in the VBM8 toolbox: http://dbm.neuro.uni-jena.de/vbm/download). If this distance was > 2SD from the sample mean from the same scanning centre then the subject was removed. Datasets were also excluded if they did not have whole brain coverage (signal present at z = − 55 (ventral bound of Crus II) or lower after normalisation). This lead to 11/28 centres (500 datasets) being excluded. A centre was also rejected if it had < 7 ASD or typically developing (TD) individuals that met our inclusion criteria. After rejection, nine centres contributed to this study with 131 ASD and 169 TD datasets. We subsequently matched the two groups for age, full scale IQ (FIQ), and head movement. This left 130 ASD and TD individuals in each group. Group demographics are reported in Table 1, and histograms are shown in Supplemental Fig. 1.

### 2.2 Pre-processing

Initial pre-processing was conducted in SPM8 (www.fil.ion.ucl.ac.uk/spm). Structural Images were first coregistered to the T1 template before the New Segmentation toolbox was used to segment the data into GM, white matter (WM), and cerebro spinal fluid (CSF) images ready for input to the DARTEL toolbox (Ashburner, 2007). The DARTEL toolbox was used to create a study specific template given that the average age of participants was 14 years ± 4 years, and as such the MNI template (generated using 18–30 years old brains) would not be appropriate (Wilke et al., 2008). The discrepancy between the MNI template and the DARTEL template used in this study are visualised in Supplemental Fig. 2. Functional images were coregistered to the individual structural images, realigned, normalized to the DARTEL template space (resliced to 3 × 3 × 3 mm), and smoothed with an 8 mm kernel. All analyses were conducted in DARTEL template space and the final results were warped into MNI space for generating figures and anatomical localization guided by the Anatomy toolbox (Eickhoff et al., 2006, Eickhoff et al., 2007, Eickhoff et al., 2005).

Further data pre-processing was conducted using in-house scripts written using MATLAB (MathWorks, Natwick, MA). Data were ‘scrubbed’ to remove bad datapoints (> 0.5 mm FD or > 0.5% differential spatial variance - DVARS; Power et al., 2012), and filtered in the band 0.009–0.2 Hz. Typically, resting state studies ignore oscillations > 0.1 Hz, however, studies by Baria et al. (2011), and Balsters et al. (2013) have demonstrated that signals between 0.1 and 0.2 Hz contain physiologically relevant information that can often be used to distinguish between clinical populations. Adequately correcting for head motion artifact has proven to be an essential step in RS-fMRI analyses, especially in investigations of ASD (Deen and Pelphrey, 2012, Tyszka et al., 2014). Based on Yan et al. (2013), and Satterthwaite et al. (2013), we modelled head movement using the Friston 24-parameter approach (Friston et al., 1996) to remove potential residual head motion signal (6 original regressors generated during realignment, 6 time shifted regressors, and both of these squared) along with the first 3 principle component time series extracted from individual WM and CSF masks (Chai et al., 2012).

### 2.3 Hierarchical clustering

In order to partition the cingulate cortex into subregions with unique connectivity fingerprints we began by creating a mask of the cingulate cortex using the Harvard-Oxford cortical atlas. Masks of the anterior cingulate, posterior cingulate, and paracingulate cortex were thresholded at > 25% and summed to create a mask of the cingulate cortex. Vogt et al. (2005), Shackman et al. (2011), Amiez and Petrides (2012), and Amiez et al. (2013) have highlighted that the presence or absence of the paracingulate sulcus can lead to a significant dorsal shift in Area 32′. We therefore chose to include the paracingulate cortex in order to make sure that Area 32′ was not excluded in subjects who have a prominent paracingulate sulcus. This mask was then warped into DARTEL space. To establish connectivity fingerprints for each subject we separated the RS-fMRI time-courses of the voxels belonging to the cingulate mask and those belonging to all GM voxels in the brain (including the original cingulate mask). We then calculated the cross correlation matrix between these two sets of time-courses, and transformed it to *z*-values using the Fisher's *r*-to-z transformation. Each column of this cross correlation matrix reflected the connectivity of a voxel within the cingulate mask with all GM voxels in the brain. We then performed a fixed-effects analysis of the matrices either separately for the ASD and TD groups, or for both groups combined. The resulting matrix was transformed back to correlation values using the inverse Fisher's transformation and used as input to a hierarchical clustering algorithm (average linkage). The resulting dendrogram was cut at multiple arbitrary uniform levels. Each level could potentially result in a different number of identified clusters. In order to determine which cut level was most appropriate we used the silhouette measure (Rousseeuw, 1987) to quantify to what extent the results derived from the group corresponded to individual data. The silhouette value assesses cluster separation by measuring how similar a voxel is to other voxels in the same cluster compared to voxels in the nearest cluster, thus maximizing within cluster similarity and between cluster differences. A silhouette value was generated for each cut level of the dendrogram for each individual subject and a *t*-test was used to determine which cut levels resulted in the highest silhouette values (cut levels most representative across individuals). We derived a plot showing the silhouette t-scores for each dendrogram cut level, and isolated local maxima as potential optimal solutions for the hierarchical clustering. The voxels belonging to a given cluster were mapped back in the brain space to generate seeds to be used in further analyses. Single-subject clustering solutions were generated by running the same hierarchical clustering procedure on individual correlation matrices and cutting the dendrogram at a position equaling the number of clusters provided by the previous group-level estimates. Finally, we used a procedure described in Mantini et al. (2013) to assess the spatial correspondence between clusters derived at the group-level and those derived from individual subjects. We used the Dice similarity measure (Dice, 1945) to compare the entire set of group-level clusters (i.e. 6 clusters) to the clusters derived for an individual (also 6 clusters). The matrix of Dice similarity values (i.e. spatial overlap between every group-level cluster and every individual-level cluster) was input into a hierarchical clustering algorithm (average linkage). After the creation of the dendrogram, we selected the cutoff value for the graph that yielding the maximum number of two-element clusters (i.e. a match between one group-level and one individual cluster). Note that this method does not enforce a minimal overlap cutoff value between a group cluster and the individual cluster solution but rather provides an overall optimal match. The Dice similarity values for each cluster are included in Table 2.

### 2.4 Seed-to-voxel analyses

Seeds generated through hierarchical clustering were used to establish seed-to-voxel connectivity fingerprints and subsequently group differences in connectivity. First, a timecourse was extracted for each seed region (averaged across voxels within the seed) and this timecourse was correlated with all GM voxels in the brain. Individual correlation maps were Fisher's *r*-to-z transformed and fed into a General Linear Model (GLM) with 10,000 permutations in Randomise (http://fsl.fmrib.ox.ac.uk/fsl/fslwiki/; (Jenkinson et al., 2012, Winkler et al., 2014)). The GLM included scanning centre, mean FD, Full scale IQ, age (log transformed to account for skewed distribution), Group × Age interaction, individual GM images, and Group × GM interaction. Individual GM images were incorporated into the GLM as voxel-dependent regressors, i.e. the GM regressor of the GLM changed for each voxel analysed to reflect GM values within the same voxel (Oakes et al., 2007). To correct for multiple comparisons at the cluster-level, we employed Gaussian random field theory, voxel-level Z > 3.1, cluster-level *p* < 0.05 FWE, based on Woo et al. (2014).

### 2.5 Meta-Analytic Connectivity Modelling

The BrainMap database (www.brainmap.org) (Fox and Lancaster, 2002, Laird et al., 2011, Laird et al., 2009a, Laird et al., 2005) was employed for the retrieval of relevant neuroimaging experiments. For our analysis, only whole brain studies of healthy subjects reporting activation in standard stereotaxic space were considered. All experiments that investigated age, gender, handedness, training effects, or involved a clinical population were excluded. As the first step of the analysis we identified (separately for each seed region) all experiments that featured at least one focus of activation within the respective seed (MNI space). In order to facilitate such filtering, coordinates from Talairach space were converted into MNI coordinates by using Lancaster transformation (Lancaster et al., 2007). Then, all experiments activating the currently considered seed were identified. The retrieval was solely based on reported activation coordinates, not on any anatomical or functional labels.

Functional connectivity of the different seeds was evaluated using Meta-Analytic Connectivity Modelling (MACM) (Robinson et al., 2012, Robinson et al., 2010). MACM assesses which brain regions are co-activated above chance with a particular seed region in functional neuroimaging experiments (Balsters et al., 2014, Eickhoff et al., 2010, Laird et al., 2009b). MACM first identifies all experiments in a database that activate a particular brain region (as described above) and then tests for convergence across (all) foci reported in these experiments. Since experiments are selected by activation in the seed, highest convergence will be observed within that region. Significant convergence of the reported foci in other brain regions, however, indicates consistent co-activation, i.e., task-based functional connectivity with the seed. The whole brain peak coordinates of the identified experiments were downloaded from BrainMap database for each seed region. Coordinates were analysed with the modified activation likelihood estimation (ALE) algorithm (Eickhoff et al., 2012, Eickhoff et al., 2009) to detect areas of convergence. This approach models each focus as a Gaussian distribution reflecting empirical estimates of the uncertainty of different spatial normalisation techniques and intersubject variability as a function of the number of subjects. Modelled activation (MA) maps were calculated for each experiment by combining the Gaussian distributions of the reported foci (Turkeltaub et al., 2012), taking the union across these voxel-wise ALE scores that describe the convergence of results at each particular location of the brain. To distinguish ‘true’ convergence between studies from random convergence, i.e., noise, in the proposed revision of the ALE algorithm (Eickhoff et al., 2012), ALE scores are compared to an analytical null-distribution reflecting a random spatial association between experiments (Eickhoff et al., 2012, Turkeltaub et al., 2012). The *p*-value of an observed ALE is then given by the proportion of this null-distribution (precisely, its cumulative density function) corresponding to equal or higher ALE values. The ALE maps reflecting the convergence of co-activations with any particular seed region were subsequently thresholded at *p* < 0.05 FWE corrected and converted into *z*-scores for display.

The functional characterization of the cingulate regions was based on the ‘Behavioural Domain’ meta-data categories available for each neuroimaging experiment included in the BrainMap database. In a first step, we determined the individual functional profile of each region of interest by using the probability of a psychological process being present given knowledge of activation in a particular brain region. This likelihood P(Task | Activation) can be derived from P(Activation | Task) as well as P(Task) and P(Activation) using Bayes rule. Significance at *p* < 0.05 (corrected for multiple comparisons using False Discovery Rate (FDR)) was then assessed with a chi-squared test (Eickhoff et al., 2011, Laird et al., 2009b, Nickl-Jockschat et al., 2011). The functional characterizations generated using the BrainMap database are generated from studies in adult healthy volunteers. However, we believe this information is still relevant for the investigation of younger age-groups (children, adolescents), and also for ASD patients. Our rationale is based on two assumptions: first, we assume that spatial normalisation is sufficient to match gross morphological landmarks and effectively match structures in younger individuals to an adult template. This ensures that the probability maps are in the same space so that functional decoding in MNI space is testing equivalent anatomical structures in all subjects. Second, we believe that structure-function relationships are consistent throughout the lifespan, i.e., a particular functional area should have the same association to macroanatomical features (that drive normalisation) in children, adolescents, and adults. This allows us to characterize, in children, adolescents, and adults, the functions that elicit an activation in a given structure. We believe this is a powerful tool for supplementing task-free analyses because it can help to generate structure-function relationships. In this study the application of functional decoding using the BrainMap database allows us to assign functions to our cingulate clusters, and subsequently infer what the behavioural consequences of deviant connectivity might be.

## 3 Results

### 3.1 Clustering the cingulate cortex

#### 3.1.1 What is the optimal number of cingulate subregions?

**Fig. 2:**
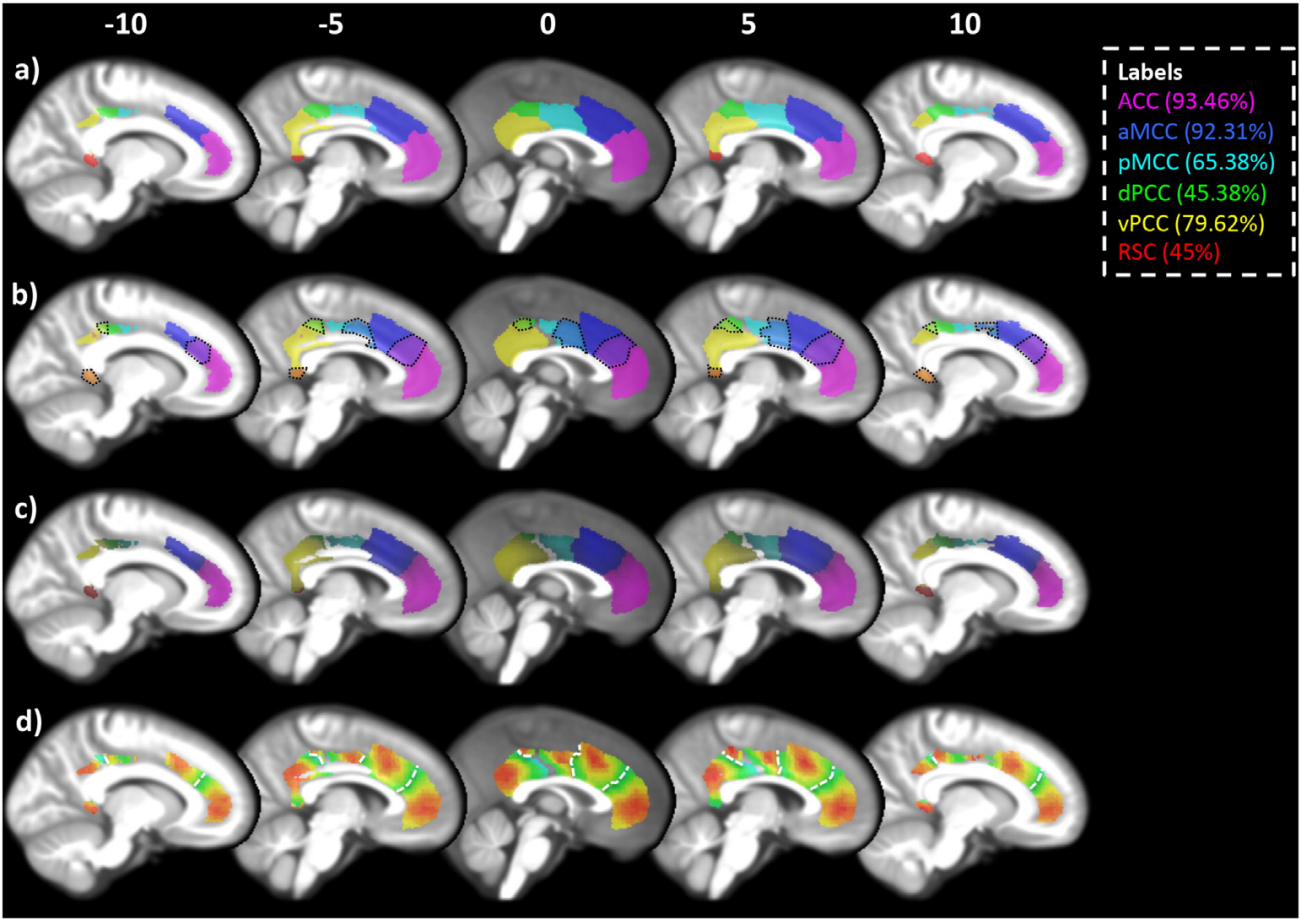
Clustering of the cingulate cortex. The cingulate cortex was partitioned into six clusters referred to throughout the manuscript as: ACC (magenta), aMCC (blue), pMCC (cyan), dPCC (green), vPCC (yellow), and RSC (red). The legend shows these labels along with the single-subject reproducibility of each cluster, i.e. the ACC cluster was present in 93.46% of subjects. A) The clustering of the cingulate cortex based on an average connectivity matrix from all participants. B) Probability maps for each cluster thresholded at 25%. The black dotted outlines show regions of overlap between clusters. C) Probability maps thresholded using a winner-takes-all approach. In the case of overlapping voxels, the voxel with the higher probability is assigned, i.e. if the voxel has a 25% probability of being ACC and 30% of being aMCC the voxel was assigned to the aMCC cluster. D) Heat maps showing the probabilities for each cluster, red being high probability and green being low. The scale of each heat map was adjusted based on the maximum of each cluster. This gives a more accurate visualisation of the variability within each cluster. White dotted lines mark the boundaries between clusters. Probability maps are available for download at http://www.ncm.hest.ethz.ch/downloads/data.html (For interpretation of the references to colour in this figure legend, the reader is referred to the web version of this article.)

First, both ASD and TD subjects RS data were input as one group into the hierarchical clustering algorithm. This revealed two clustering solutions with significant across-subject similarity. The first solution separated the posterior cingulate cortex (PCC) from the anterior cingulate and paracingulate gyrus (supplemental Fig. 3). The second solution subdivided the cingulate cortex into six clusters (Fig. 2). This second solution is more anatomically plausible based on previous studies of cytoarchitecture and connectivity-based parcellations (Beckmann et al., 2009, Neubert et al., 2015, Palomero-Gallagher et al., 2009, Torta et al., 2013). We will also demonstrate later (Section 3.2.2) that the subdivisions of the ACC present in the six cluster solution have unique connectivity fingerprints and behavioural profiles, further supporting the use of this higher order clustering solution. Although ASD and TD individuals were treated as one group, silhouette values were generated for each individual. This allowed us to check for group differences in silhouette values to determine whether the group mean was a better representation of one group over another. There were no significant differences in the silhouette value between groups (ASD: 0.157 ± 0.117; TD: 0.157 ± 0.135; t(258) = 0.0476, *p* = 0.96).

**Fig. 3:**
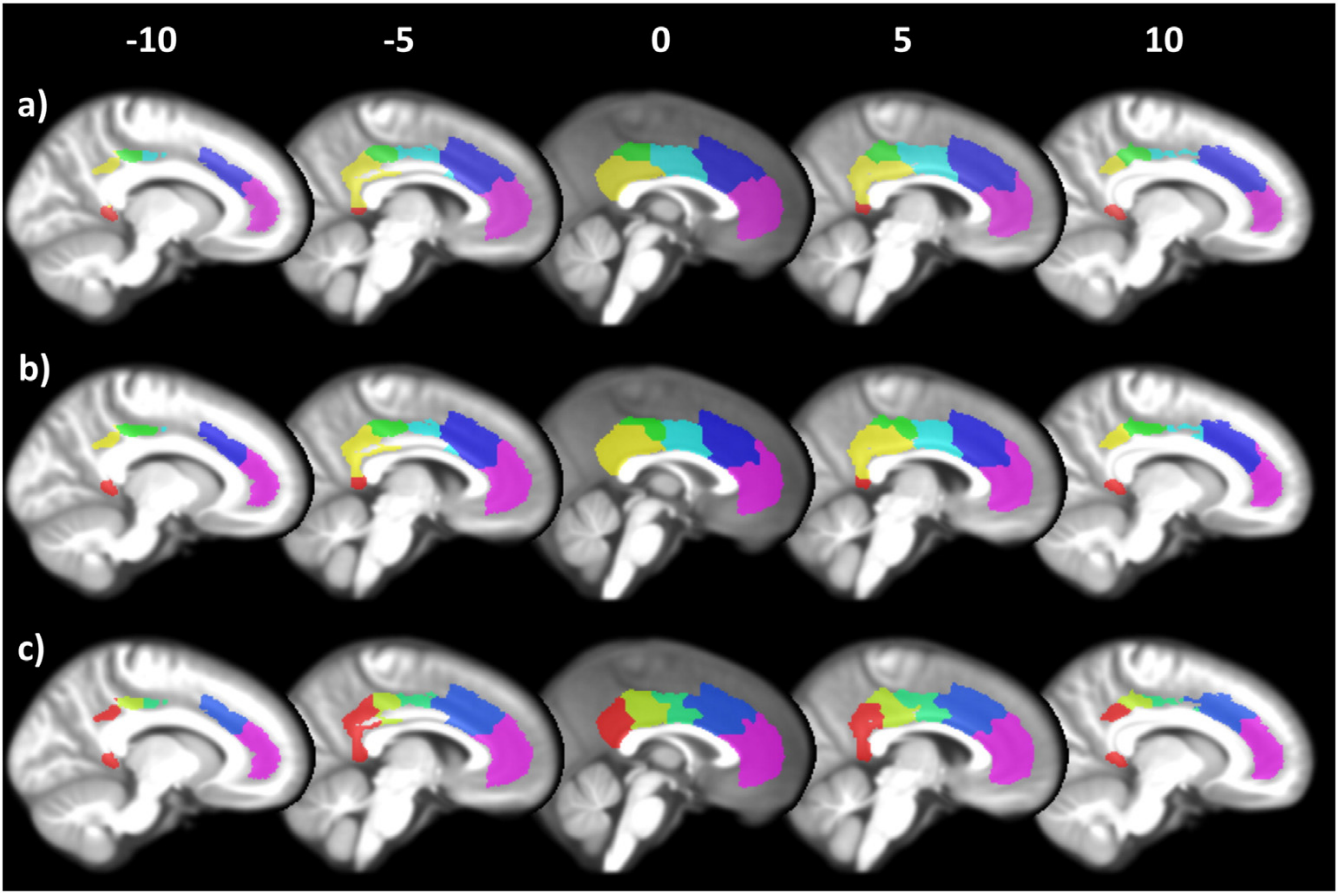
Clustering solutions for (a) all subjects, (b) ASD only, (c) TD only. All subjects and ASD subjects showed a highly similar 6 cluster solution, whereas TD only subjects showed 5-cluster solution where the vPCC (yellow in a and b) and RSC (red in a and b) were merged. Apart from this difference all other clusters in the TD clustering solution were similar to the ASD and all subject solution.

ASD and TD groups were also analysed separately to determine whether there were different optimal solutions for each group (Fig. 3). As in the previous step, silhouette values were generated for each individual, however, in order to allow for different solutions for each group we created separate dendrograms. Thus cutting the dendrograms at the same arbitrary point could result in different clustering solutions for each group. This approach yielded a six-cluster solution in ASD and a five-cluster solution in the TD group (Fig. 3). The six-cluster solution for the ASD group was almost identical to the six-cluster solution generated using all subjects. The five-cluster solution for the TD group was highly similar to the six-cluster solution, however, the ventral posterior cingulate cluster (Fig. 2a, yellow) and the retrosplenial cortex (Fig. 2a, red) were merged together. Given the high degree of similarity between these group specific solutions, and their similarity to the solution generated based on all subjects (which showed no group differences, *p* = 0.96) the six-cluster solution generated from both groups was chosen for subsequent analyses.

#### 3.1.2 How replicable are subregions at the single subject level?

Dice similarity was used to assess how often each of the six clusters in the group mean were replicated across individuals, i.e. if a cluster at the individual level matched a cluster at the group mean level it was marked as 1, otherwise it was marked as 0. When a group level cluster was not found at the individual level it was mostly because the cluster was subsumed by an adjacent cluster. This is similar to the example given previously where the ventral posterior cingulate cortex and retrosplenial cortex were merged. The labels used to describe these clusters were taken from the four-region model of the cingulate cortex (Palomero-Gallagher et al., 2009, Vogt, 2009). The anterior cingulate cortex (ACC: Fig. 2a magenta) was the most replicable across subjects (93.46% of individual subject clustering solutions showed a cluster that was significantly similar in position and shape to the group-based ACC). Replicability of the other clusters ranged from 92.31% to 45% (see Fig. 2a and Table 2). Chi squared tests found no differences in cluster matching between groups for any cluster (*p* > 0.44), further suggesting that the organisation of cingulate connections is highly similar in ASD and TD individuals. Based on the similarity between groups in silhouette values and single-subject replications of the clustering solution, it is unlikely that the six-cluster solution introduces bias against TD individuals.

Probability maps for each cluster were created by binarising and summing clusters from each individual subject (available for download at http://www.ncm.hest.ethz.ch/downloads/data.html). Fig. 2b shows probability maps thresholded at > 25%. It is clear that there are a number of overlapping voxels between clusters (black dotted lines highlight areas of overlap). We therefore used a winner-take-all approach and assigned overlapping voxels to the cluster with the greater probability. For example, if there was an overlapping voxel between the ACC and aMCC, and it had a 53% probability of being ACC and 47% probability of being aMCC, then the voxel was assigned to the ACC cluster. The number of overlapping voxels with the exact same probability was very low (typically below < 0.3%, but in the case of vPCC and dPCC this was a little higher at 1.2%). The winner-take-all probability maps are shown in Fig. 2c and details of the strength and size of the probability maps are included in Table 2. Fig. 2d shows the heat maps for the probability of each cluster.

### 3.2 Function and connectivity of cingulate clusters

#### 3.2.1 Is there an overlap between task-independent and task-dependent connectivity fingerprints?

**Fig. 4:**
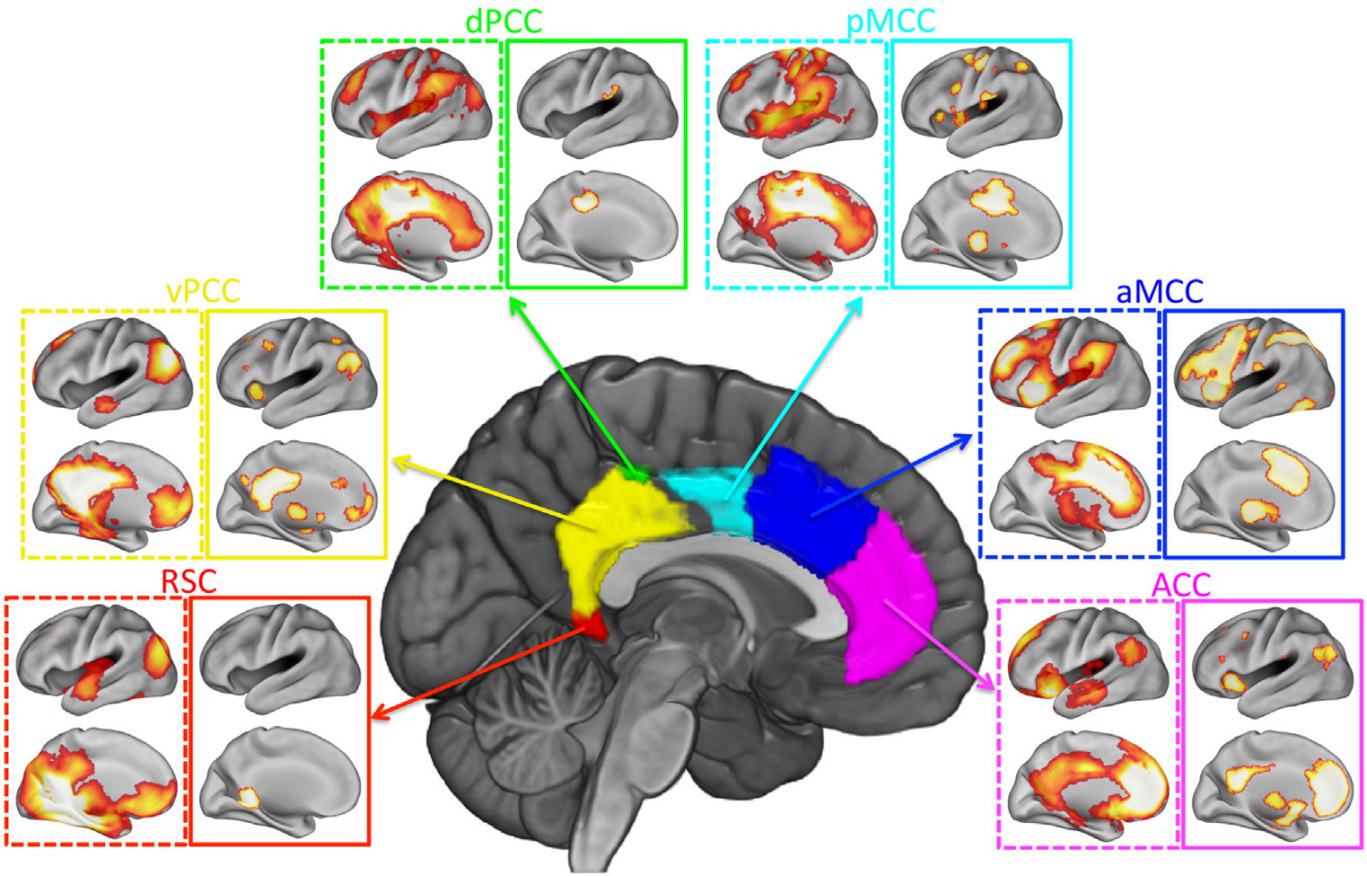
Comparison between resting state and MACM connectivity fingerprints. For each cluster, resting state (left, dashed line box) and MACM (right, solid line box) connectivity maps are shown side-by-side. Both resting state and MACM maps were corrected for multiple comparisons (FWE, *p* < 0.05). Only the left hemisphere is presented for each modality, however the left and right hemisphere showed highly similar connectivity patterns. The colour code for boxes corresponds to the colour code established in Fig. 2.

Fig. 4 shows the RS-fMRI (task-independent) connectivity fingerprint for each cluster (dashed box), next to the task-dependent connectivity fingerprint (solid box) as established through MACM. Generally, RS-fMRI connectivity revealed more widespread connectivity fingerprints than MACM, which is not surprising given that the input data for MACM are activation maxima taken from reported literature. Nevertheless, we found consistent overlaps between task-independent and task-dependent connectivity maps for most cingulate subregions. We used Dice similarity and hierarchical clustering to compare task-independent and task-dependent connectivity fingerprints (Mantini et al., 2013). Dice similarity showed that there was a significant overlap in the connectivity fingerprints for the ACC (29% overlap), aMCC (40% overlap), pMCC (18% overlap) and the vPCC (20% overlap). Task-dependent and task-independent connectivity fingerprints for the dPCC (6% overlap) and the RSC (3% overlap) were not significantly similar. Anatomical details of the overlapping regions in each connectivity map are provided in supplemental results. Given that the task-independent connectivity fingerprints included both ASD and TD subjects, whilst task-dependent connectivity maps from MACM only included healthy subjects, we repeated the Dice similarity using the task-independent connectivity fingerprints generated from TD subjects only. The results were almost identical to the previous results (Significant overlap: ACC 28%; aMCC 40%; pMCC 19%; vPCC 19%; non-significant overlap: dPCC 6%; RSC 3%).

#### 3.2.2 Functional characterization of cingulate clusters

**Fig. 5:**
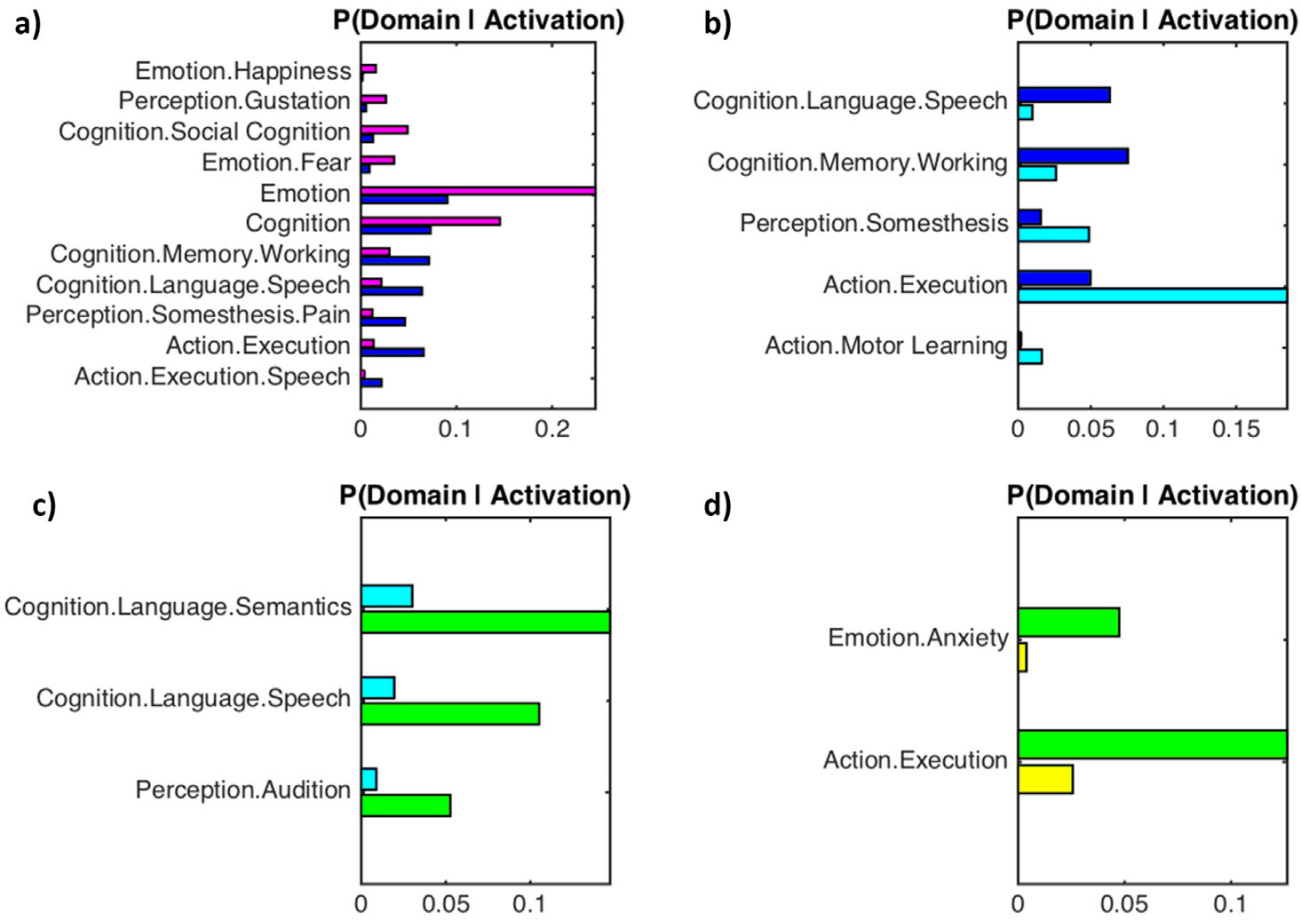
Functional characterization of cingulate clusters: bar plots show the reverse inference Bayesian probabilities (i.e. the likelihood of a behavioural domain given the location of the activation cluster) for behavioural domains associated with each cluster. Behavioural domains are contrasted with adjacent clusters and corrected for multiple comparisons (FDR, *p* < 0.05). Thus a larger bar indicates a behavioural domain is more likely to elicit activity in a specific cluster. The colour of each bar corresponds to the colour code established in Fig. 2. A) ACC (magenta) compared to aMCC (blue). B) aMCC (blue) compared to pMCC (cyan). C) pMCC (cyan) compared to dPCC (green). D) dPCC (green) compared to vPCC (yellow). (For interpretation of the references to colour in this figure legend, the reader is referred to the web version of this article.)

In order to illustrate the functional specificity of each cluster we compared behavioural domains and paradigm classes associated with each cluster contrasted against adjacent clusters. Fig. 5 shows the reverse inference Bayesian probability (i.e. the likelihood of a behavioural domain eliciting an activation within a given cluster). The likelihood of specific paradigm classes activating a given cluster are provided in supplemental Fig. 4. In summary, the ACC and vPCC are most likely to be activated by emotion and (social) cognition behavioural domains, the aMCC is most likely to be activated by attention and working memory, and the pMCC and the dPCC are most likely to be activated by movement execution, somatosensory perception and some aspects of language processing. There were no significant differences in the behavioural domains or paradigm classes between the vPCC and RSC.

#### 3.2.3 How does connectivity strength change due to the size and placement of a seed?

**Fig. 6:**
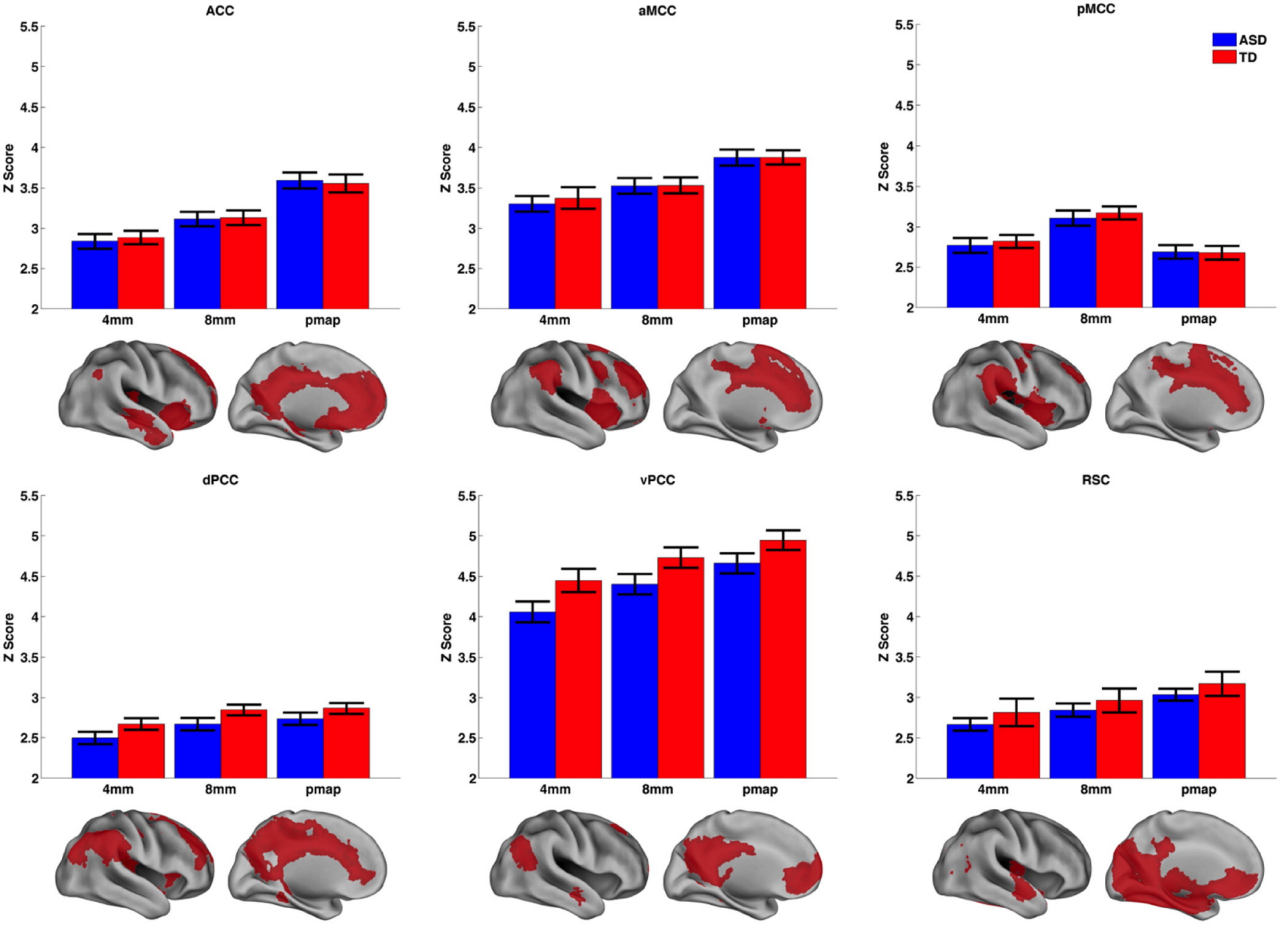
Connectivity strength for differently sized seeds. Bars show the average *z* value across voxels in each network for each different seed threshold. The network of voxels that are averaged is shown underneath each bar graph. The seeds tested included the probability maps shown in Fig. 2c, and a 4 mm and 8 mm radius sphere around the peak of the probability maps. Blue bars show ASD and red bars show TD individuals. Error bars show the standard error. (For interpretation of the references to colour in this figure legend, the reader is referred to the web version of this article.)

In order to compare with previous seed-based studies, we compared the probability maps generated in this study (Fig. 2c) with 4 mm and 8 mm radius sphere seeds centred at the peak of the probability maps. To establish network connectivity strength we averaged the *z*-values of voxels with cluster specific connectivity masks. These masks only included voxels that showed significant connectivity (FWE, *p* < 0.05) with all three seed types (i.e., mask voxels showed significant connectivity with the 4 mm sphere, 8 mm sphere, and probability maps). Fig. 6 shows bar graphs highlighting the network strength (averaged *z*-value of the voxels in the mask underneath the bar graph) for each cluster. All clusters showed a significant main effect of seed type (highest: RSC: F(2516) = 168.08, *p* < 0.001; lowest: pMCC: F(2516) = 62.23, *p* < 0.001). Fig. 6 shows that in 5/6 cases there was significantly higher network connectivity using probability maps compared to 4 mm or 8 mm spheres. There were no significant group differences or Group × Seed type interactions. This shows that the use of connectivity-based probability maps significantly increases the ability to detect connectivity networks compared to traditional approaches using smaller spheres.

**Fig. 7:**
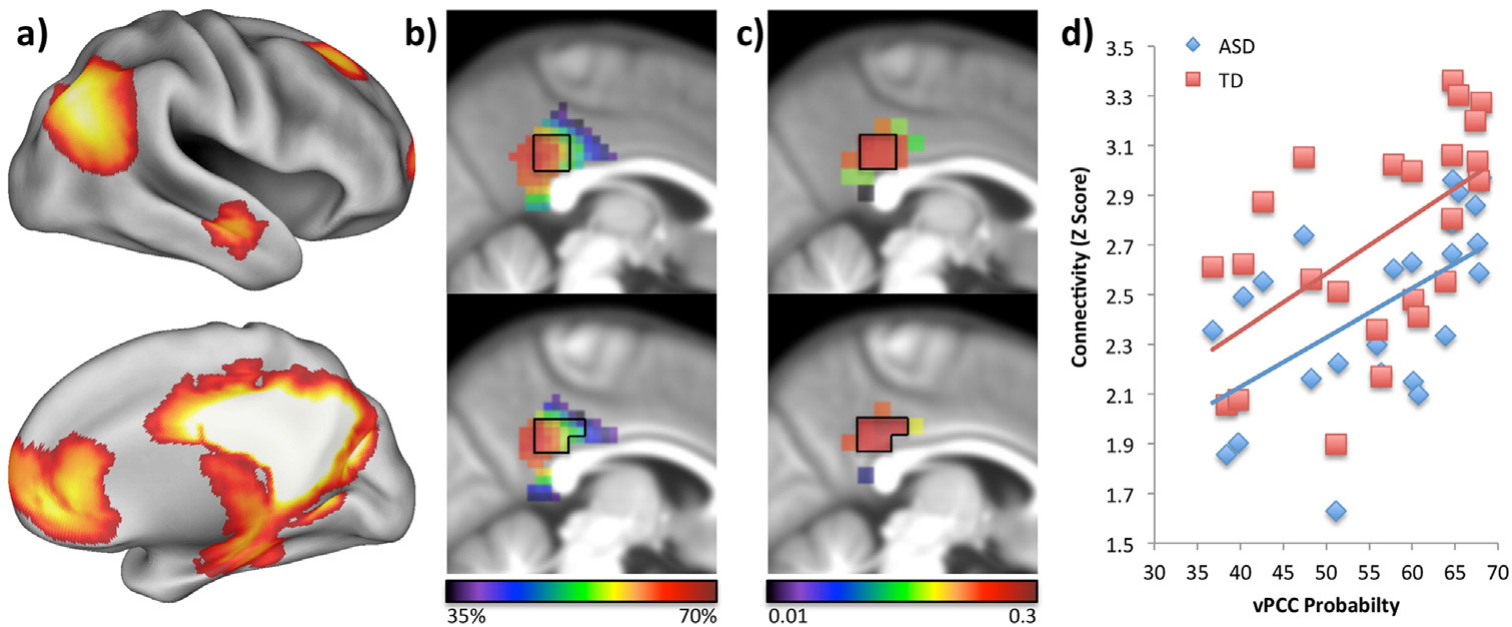
Varying seed placement and its relationship to group difference detection. A) vPCC connectivity fingerprint, putatively the DMN. B) vPCC probability map and C) group differences (effect size) at each 4 mm radius sphere. Black/purple indicates a low probability/effect size with yellow/red indicating a high probability/effect size. Slices in (b) and (c) are taken from X = − 2 (upper panel) and X = 5 (lower panel). The black outline indicates where there were significant group differences (TD > ASD). D) Scatter plot showing the correlation between network strength (Z score) and vPCC probability for each group.

Although there were no group differences, there was a trend towards a main effect of group for the vPCC seeds (*p* = 0.059). The greatest group difference appeared to be with the 4 mm sphere, so we transformed the vPCC probability map into twenty-four 4 mm spheres and assessed the variability of the group connectivity fingerprints. Fig. 7a shows the mask used to extract z-values (voxels showing significant connectivity using the winner-takes-all vPCC probability map (yellow: Fig. 2c)), and Fig. 7b shows the probability map for the vPCC cluster. Next, for each 4 mm seed region we investigated whether connectivity differed between ASD and TD groups. The ASD group exhibited generally lower connectivity (Z scores for each 4 mm sphere are graphically presented in supplemental Fig. 5), but depending on where the 4 mm sphere was placed inside the vPCC probability map, the effect size of this group difference ranged from 0 to 0.3 (Fig. 7c: black/purple/blue indicate a low effect size and yellow/red indicates a larger effect size). Spheres showing a significant group difference are outlined in black on Fig. 7b,c. Fig. 7d shows that connectivity strength strongly correlated with vPCC probability in both groups (ASD: *r* = 0.57, *p* = 0.004; TD: *r* = 0.57, *p* = 0.004). Although there was no significant relationship between effect size and vPCC probability (*r* = 0.22, *p* = 0.31), the group differences appear to be in the centre of the probability map and group differences decrease around the edges.

### 3.3 Are there differences in cingulate connectivity in ASD?

**Fig. 8:**
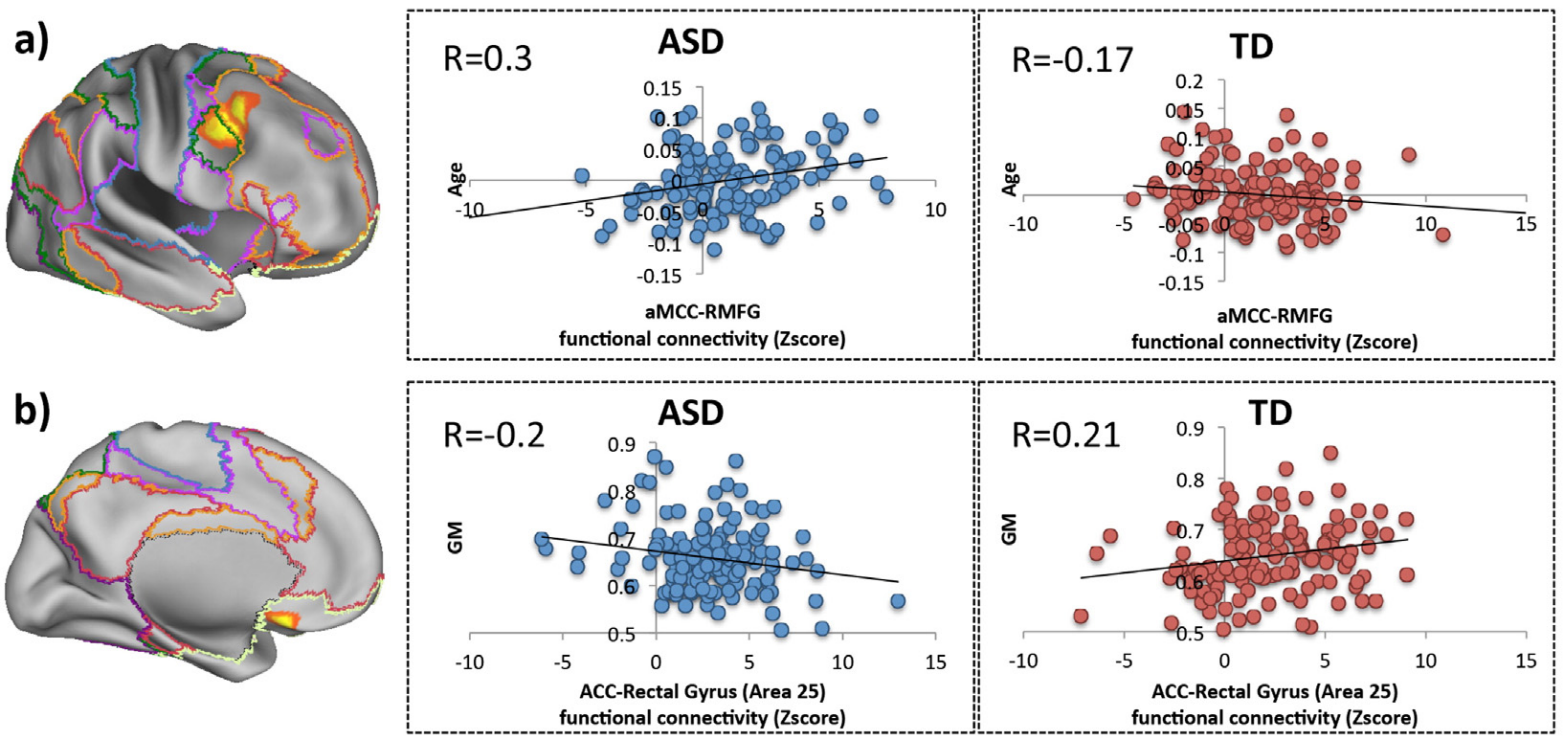
Group differences in resting connectivity. Group differences were overlaid on an inflated brain. The coloured lines highlight the boundaries of resting state networks established by Yeo et al. (2011). A) A Group × Age interaction between the aMCC and right lateral PFC. The scatter plots to the right show that connectivity between these regions increased with age in ASD (blue) but decreased with age in TD (red). B) A Group × GM interaction between the ACC and the rectal gyrus (area 25). The scatter plots to the right show that connectivity decreased as GM in the rectal gyrus increased in ASD (blue) whilst connectivity increased as rectal gyrus GM increased in TD (red). In all scatter plots the correlation values are shown in the top left corner. (For interpretation of the references to colour in this figure legend, the reader is referred to the web version of this article.)

A Group × Age interaction was present between aMCC and the right precentral/middle frontal gyrus (Fig. 8a). This interaction is due to connectivity between the aMCC and right precentral/middle frontal gyrus increasing with age in ASD (*r* = 0.3, *p* < 0.001), whereas connectivity between these regions decreased with age in TD individuals (*r* = − 0.17, *p* = 0.053). This result was significant at cluster extent Z > 3.1 (*p* = 0.029, FWE corrected) with a moderate effect size of 0.53 (Cohen's d). A Group × GM interaction was also present (Fig. 8b), with connectivity between the ACC and the Rectal gyrus (Area 25) decreasing with increasing GM in ASD (*r* = − 0.2, *p* = 0.023), whereas connectivity increased with increased GM in TD (*r* = 0.21, *p* = 0.016). This result was significant at cluster extent Z > 3.1 (*p* = 0.047, FWE corrected) with a moderate effect size of 0.54 (Cohen's d). Age and GM effects common to both groups are presented in supplemental Figs. 7–11.

## 4 Discussion

Here, we applied a new analysis pipeline to investigate brain connectivity in ASD. We used the cingulate cortex as our anatomical region of interest because it is one of the most investigated structures in ASD (Cauda et al., 2011), and a great deal is known about its organization and connectivity (Beckmann et al., 2009, Margulies et al., 2007, Neubert et al., 2015, Torta and Cauda, 2011, Torta et al., 2013, Vogt, 2009). Our hierarchical clustering approach partitioned the cingulate into regions consistent with existing models (Palomero-Gallagher et al., 2009, Torta and Cauda, 2011). The validity of our clustering solution was further supported by: (i) demonstrating an overlap between task-dependent and task-independent connectivity fingerprints, (ii) showing that clusters with distinct connectivity fingerprints participate in different behaviours, and (iii) demonstrating the robustness and replication of clustering solutions at the single subject level. We also demonstrated that using probability maps as seeds increased network strength across both groups, and that seeds placed on the boundaries of our probability maps were less likely to highlight group differences in connectivity. Our results suggest that the organization of the cingulate cortex, as well as overall cingulate connectivity strength in ASD, were more similar to TD individuals then previously suggested. We did, however, demonstrate a Group × Age interaction showing increased connectivity between the aMCC and right precentral/middle frontal gyrus with age in ASD. We also found a Group × GM interaction between the ACC and the rectal gyrus (Area 25) showing increased connectivity with rectal gyrus GM in TD individuals.

### 4.1 Dissecting Brodmann's ACC into distinct subregions

A number of studies have proposed that there is a rostral-caudal functional gradient in the frontal lobe (Badre and D'Esposito, 2009, Blumenfeld et al., 2013, Koechlin and Summerfield, 2007). Brodmann (1909) was the first to propose a rostral-caudal distinction within the cingulate cortex, specifically separating the precingulate cortex (classical ACC) and postcingulate cortex (PCC) based on the differences in their cytoarchitecture. However, more recent analyses by Vogt et al. (2005) and Palomero-Gallagher et al. (2009) have shown that Brodmann's (1909) ACC can be further separated into four subregions; subgenual and pregenual ACC, aMCC, and pMCC. Our clustering analyses provided two winning solutions; one provided an ACC cluster similar to Brodmann's ACC or the ACC mask in the Harvard-Oxford cortical atlas (see supplemental Fig. 3) whilst the other split the ACC into three distinct subregions: the pregenual ACC, aMCC, and pMCC (the subgenual ACC was missing because it was not part of the original mask). We proposed (Section 3.1.1) that the solution that separates the ACC, aMCC, and pMCC (Fig. 2) was more appropriate despite it having a lower silhouette value. Finding the “optimal” number of clusters is still an unresolved issue for connectivity-based parcellations. Eickhoff et al. (2015) comprehensively review a number of potential metrics for establishing the validity of clustering solutions, but conclude that coherence across multiple tools is the most likely to lead to the ground truth. Our rationale for choosing the second clustering solution (six cluster solution) was driven by the combination of cluster separation criteria (silhouette values) as well as two sources of external knowledge: 1) the separation of the ACC, aMCC, and pMCC conforms with more recent anatomical models of the cingulate cortex that include mylo- and cyto- and receptor architecture (Palomero-Gallagher et al., 2009, Vogt et al., 2005), and 2) the ACC, aMCC, and pMCC clusters generated in this study had unique behavioural profiles established using the Brainmap database (Section 3.2.2). Whilst there may be some circularity in selecting a winning solution based on the previous studies, the independent evidence derived from the Brainmap database strongly supports the idea that Brodman's ACC should be split into three unique subregions.

The historical legacy of Brodmann's classification has lead to the ACC being used as a “catch-all” term, often leading to the incorrect labelling of the aMCC as dorsal ACC. This mislabelling has led many to inaccurately discuss the functional properties of an aMCC result in relation to the functional and anatomical properties of the ACC (Apps et al., 2013). The results of our parcellation strongly support the distinction between ACC and aMCC based both on task-independent and task-dependent connectivity fingerprints, along with unique contributions of the ACC and aMCC to distinct behavioural domains. Consistent with Torta and Cauda (2011), we found that the ACC was involved in emotion and reward studies, whilst the aMCC showed a functional preference for working memory. A recent article by Liberman & Eisenberger (Lieberman and Eisenberger, 2015), has suggested that the aMCC is ‘selectively’ activated by pain. This assertion has lead to a number of interesting follow-up discussions online between Liberman & Eisenberger and Shackman, Yarkoni, and Wager (#cingulategate). In line with Lieberman and Eisenberger (2015), we found that the most common paradigm for eliciting activity in the aMCC was pain monitoring/discrimination (see supplemental Fig. 4). However, this was only just larger than the delayed match-to-sample paradigm. Moreover, when we analysed which behavioural domains are likely to elicit activity in the aMCC we found a higher probability for working memory than for pain perception (Fig. 4a). These differences between our findings and the findings of Lieberman and Eisenberger (2015) might result from inclusion of the paracingulate cortex in our initial mask, whereas Lieberman and Eisenberger (2015) only considered the ACC and assumed that regions dorsal to this are more likely to reflect the pre-supplementary motor area (preSMA). Unfortunately, meta-analytic approaches lack information about individual anatomical variation, so it is not possible to confirm whether our initial mask was overly generous and includes sections of preSMA, or whether Lieberman and Eisenberger (2015) have been too conservative and ignored Area 32′. The point we wish to highlight here is that there are clear functional differences between the ACC and aMCC, which is also supported by Liberman & Eisenberger (2015). Given that group differences were found in both the ACC and aMCC, we would suggest that disruptions to these cingulate subregions have unique behavioural outcomes (see Section 4.3).

As well as separating the ACC and aMCC, we also found a separate cluster for pMCC. Similar to a number of other structures (Johansen-Berg et al., 2004, Picard and Strick, 2001), the VAC line perfectly separates the aMCC and pMCC (supplemental Fig. 12). The pMCC showed the strongest relationship to the motor control behavioural domain. This finding is supported by both task-independent and task-dependent connections with a motor network consisting of dorsal portions of the primary motor cortex (area 4p), and the second somatosensory area (S II). The behavioural profile also shows that the pMCC cluster contributes to action execution tasks, and paradigms activating this region included finger tapping and tactile discrimination. The separation between the aMCC and pMCC is consistent with the rostral-caudal gradient of motor function within the cingulate proposed by Picard and Strick (2001). However, Picard and Strick (2001) suggested three regions (anterior rostral cingulate zone, posterior rostral cingulate zone, and caudal cingulate zone) related to conflict monitoring, action selection, and action execution respectively. Our results, along with other clustering studies using MACM (Hoffstaedter et al., 2013) and diffusion weighted images (Beckmann et al., 2009) suggest a merging of the posterior rostral cingulate zone and caudal cingulate zones, both of which would be involved in action selection and execution.

### 4.2 The impact of seed size and placement in the vPCC

The ACC and vPCC presented with significantly similar behavioural profiles, even though they have unique connectional fingerprints. Given their location and similar behavioural profiles, it is likely that these are the anterior and posterior nodes of the DMN. Given the link between the DMN and social cognition, this has probably been the most investigated network in ASD. A number of studies have shown reduced connectivity within the DMN in ASD (Assaf et al., 2010, Di Martino et al., 2013 Di Martino et al., 2009, Kennedy et al., 2006, Kennedy and Courchesne, 2008a, Kennedy and Courchesne, 2008b ; Spencer et al., 2012, Starck et al., 2013, Washington et al., 2014), and in this study we found a trend towards underconnectivity of the vPCC network (putatively the DMN) in ASD, although this did not reach significance (network strength based on the vPCC probability map: t(258) = − 1.65, *p* = 0.1). However, there are a number of previous studies that also fail to find differences in the DMN when different analytical steps are taken, for example differences in low-pass filtering, task regression, or head movement correction (Müller et al., 2011, Tyszka et al., 2014). There have also been several recent investigations of functional connectivity in ASD suggesting that careful characterization of participant age is necessary in order to avoid obscuring developmental group differences (Abbott et al., 2015, Nomi and Uddin, 2015, Uddin et al., 2013). One issue that has not previously been discussed is the placement of smaller seeds. It is very typical to use 4 mm radius spheres to generate time series of interest for RS-fMRI analyses, however, transforming our vPCC probability map into smaller 4 mm radius spheres showed the variability present in this approach. Within one probability map, group difference effect size varied from 0 to 0.3 (ES > 0.25 showed significant differences), and the likelihood of finding a connectivity difference between the ASD and the TD group decreased when the seed was moved to the edges of the vPCC probability map. Although there was no significant relationship between effect size and vPCC probability, both groups showed a highly significant relationship between network strength and vPCC probability (Fig. 7d). Given the variability present in the spatial topographies of some resting state networks across individuals with ASD (Hahamy et al., 2015) this connectivity-based parcellation approach can help to reduce the likelihood of Type II errors and help to produce more robust and informative biomarkers for clinical RS-fMRI.

### 4.3 Opposing developmental trajectories in cingulate connectivity between ASD and TD individuals

Although there were no significant group differences in cingulate connectivity, we did find group interactions with age, and GM. Specifically, connectivity between the aMCC cluster and right lateral PFC increased with age in ASD, but decreased in age in controls. Increased connectivity between the lateral PFC and aMCC is consistent with postmortem studies of ASD (Zikopoulos and Barbas, 2013 , 2010). Zikopoulos and Barbas, 2013, Zikopoulos and Barbas, 2010hite matter below the ACC/aMCC (area 32). Zikopoulos and Barbas (2010) also found a decrease in the ratio of calbindin (CB) and parvalbumin (PV) inhibitory interneurons in the lateral PFC (area 9). CB neurons within area 9 of the LPFC have been shown to be targeted preferentially by ACC/aMCC pathways (Medalla and Barbas, 2009), suggesting that connectivity between the lateral PFC and ACC/aMCC is likely to be increased in ASD. Zikopoulos and Barbas (2013) propose that this pathway facilitates gain modulation during attentional processes, and that attentional deficits seen in ASD, such as excessive focusing on one stimulus or thought and the inability to disengage and attend to other stimuli flexibly, could be due to increased connectivity between the aMCC and lateral PFC. Our findings add support to this hypothesis by suggesting that there may be an additional developmental dimension to this process given that ASD and TD connectivity trajectories appear to differ dramatically with age. Further task-based studies are necessary in order to disentangle the relationship between ASD, age, and attention.

We also showed a Group × GM interaction in connectivity between the ACC and the rectal gyrus (Area 25). Specifically, we demonstrated in controls that as GM increased in the rectal gyrus, so did its connectivity with the ACC. However, ASD individuals showed a decrease in ACC-rectal gyrus connectivity when GM in the rectal gyrus increased. A recent study by Palomero-Gallagher et al., (Palomero-Gallagher et al., 2015) showed that the ACC and Area 25 have similar functional profiles (both were associated with emotion and reward paradigms). The link between social and emotional processing, and reward has been highlighted in the Social Motivation Theory of ASD (Chevallier et al., 2012, Dawson et al., 2012), which suggests that the value of social and emotional stimuli are down-regulated in individuals with ASD. A meta-analysis of social and non-social paradigms reported that individuals with ASD showed reduced activation in the ACC during social paradigms (Di Martino et al., 2009). This reported activation falls close to the peak of our ACC probability map (79% probability). The relationship between increased connectivity and decreased GM in ASD is not necessarily intuitive, however, Tang et al. (2014) previously found that an increase in GM volume in ASD can be explained by a lack of dendritic spine pruning in individuals with ASD. This decrease in dendritic spine pruning was linked to the mammalian target of rapamycin (mTOR) signaling, and rodents with overactive mTOR signaling also show ASD-like social behaviours (Yizhar et al., 2011). Thus an increase in GM volume (or lack of dendritic spine pruning) can actually be linked to poorer social behavior. Even though it is highly speculative at this point we suggest that this increase in GM volume may be indicative of a lack of dendritic pruning, which in turn may have a knock-on effect on connectivity with the ACC, and in turn influence social behaviour.

## 5 Conclusions

Whilst RS-fMRI has the potential to be a powerful tool for developing neurophenotypes, there are still methodological issues that reduce its utility as a clinical tool. Our novel data-driven approach for generating seeds based on connectivity fingerprints circumvents potential methodological issues regarding seed placement and significantly improves network detection. Using this approach, we demonstrated that the organisation of the cingulate cortex is far more similar in ASD then previously suggested (connectivity based probability maps of the cingulate cortex in both ASD and TD groups are available for download at http://www.ncm.hest.ethz.ch/downloads/data.html). Although, perturbed connectivity was present in two distinct cortico-cingulo circuits, which may lead to commonly reported ASD deficits in attention and social interaction. Future studies should investigate other cortical and subcortical regions of interest, which may provide more reliable connectivity-based biomarkers of ASD.

## Figures and Tables

**Table 1:** Participant demographic.

	Controls				ASD					
	Min	Max	Mean	SD	Min	Max	Mean	SD	t value	*p* value
Age (years)	7.26	31.78	14.87	4.90	7.15	32.00	13.94	4.30	1.62	0.11
IQ	83.00	135.00	109.40	11.55	81.00	136.00	106.82	12.86	1.70	0.09
Framewise displacement (mm)	0.08	0.31	0.17	0.05	0.06	0.28	0.17	0.05	0.07	0.95

**Table 2:** Peak coordinates, size, and strength of probability maps. The peak co-ordinates are given in MNI space. The max value reflects the peak value of the probability map. Dice similarity values indicate the average spatial overlap between a group-level cluster and an individual-level cluster.

	Peak			Max	Cluster size	Dice similarity		
x	y	z	(%)	(mm^3^)	Mean	SD	% Matches
ACC	3	44	3	82	25,150	0.67	0.15	93.46
aMCC	6	19	39	77	23,314	0.62	0.14	92.31
pMCC	10	− 8	41	47	7392	0.51	0.14	65.38
dPCC	8	− 33	45	35	3389	0.53	0.17	45.38
vPCC	5	− 46	25	70	15,821	0.65	0.16	79.62
RSC right	13	− 47	0	42	1242	0.75	0.19	45.00
RSC left	− 14	− 41	− 3	42	983

## Appendix A Supplementary data

Supplementary materials. Image 1
