# Supplementary material for: Connectivity-based parcellation increases network detection sensitivity in resting state fMRI: An investigation into the cingulate cortex in autism
Source: Neuroimage Clin. 2016 Mar 25;11:494–507. doi: 10.1016/j.nicl.2016.03.016 (PMC4832089; doi:10.1016/j.nicl.2016.03.016)
Supplement: Supplementary file 1 — Supplementary materials. [file mmc1.docx]

Connectivity-based parcellation increases network detection sensitivity in resting state fMRI: An investigation into the cingulate cortex in Autism

**Supplemental Materials**

Joshua H. Balsters*^1^, Dante Mantini ^1,2,3^, Matthew A. Apps ^2^, Simon B. Eickhoff ^4,5^, & Nicole Wenderoth ^1,3^

^1^ Neural Control of Movement Lab, Department of Health Sciences and Technology, ETH Zurich, Switzerland

^2^ Department of Experimental Psychology, University of Oxford, Oxford OX1 3UD, UK

^3^ KU Leuven, Kinesiology and Rehabilitation Sciences, Research Center for Movement Control and Neuroplasticity, Heverlee, Belgium

^4^ Institute of Neuroscience and Medicine (INM-1), Research Center Jülich, Germany

^5^ Institute of Clinical Neuroscience and Medical Psychology, Heinrich-Heine University Düsseldorf, Germany

**Corresponding Author:**

Dr Joshua Henk Balsters

Neural Control of Movement Lab

Department of Health Sciences and Technology

ETH Zurich,

Switzerland

E-Mail: Joshua.balsters@hest.ethz.ch

Telephone: +41 44 63 56101

# Supplemental Results

## Group differences in grey matter (GM)

First, we ran a VBM analysis to establish if there were differences in GM that could account for group differences, or a lack of group differences, in resting connectivity. Here, we used a GLM that included scanning centre, age (log transformed), and FIQ as regressors of no interest. Supplemental figure 7a shows group differences in GM where ASD individuals had significantly more GM compared to controls. There were no voxels showing a significant difference in the opposite direction. In order to relate differences in GM to resting state networks the boundaries of resting state networks generated by Yeo et al [2011] were also drawn on the figure. In keeping with Yeo et al [2011] , dark purple outlines the visual network, blue outlines the somatomotor network, green outlines the dorsal attention network, violet outlines the ventral attention network, cream outlines the limbic network, orange outlines the frontoparietal network (FPN), and red outlines the DMN. Most group differences in GM appear to be in the superior and inferior temporal gyri, along with the orbitofrontal cortex. We quantified the overlap between significant differences in GM and the networks from Yeo et al [2011] illustrating how these GM voxels were distributed across the different resting state networks (Supplemental figure 7b) as well as how many voxels of a specific network exhibited a GM group difference (Supplemental figure 7c). The greatest differences in GM were present in the limbic network (34.05% of the limbic network), followed by the sensorimotor network (12.69% of the sensorimotor network), and the DMN (12.47% of the DMN network). In a number of cases, it is clear that differences in grey matter conform to the functional boundaries established by Yeo et al [2011]. For example, the ventral view of GM differences presented in supplemental figure 7a shows that significant differences in the orbitofrontal cortex follow the boundary of the limbic network (cream line) and do not cross over into the FPN (orange line).

## Overlap between task-independent and task-dependent connectivity fingerprints

For the ACC cluster, connectivity fingerprints overlapped in the left middle frontal gyrus (Area 8b), bilateral anterior insula, bilateral nucleus accumbens, precuneus, and the left angular gyrus (PGp). The aMCC connectivity fingerprints also showed overlap in the bilateral anterior insula, as well as bilateral MFG (Area 9/46d), bilateral precentral gyrus, bilateral anterior putamen, bilateral prefrontal thalamus, and bilateral intraparietal sulcus (hIP2). The pMCC connectivity fingerprints showed overlap between bilateral postcentral gyrus (Area 4p), bilateral posterior insula overlapping with the rolandic operculum (OP4), and bilateral secondary somatosensory area (SII). The dPCC connectivity fingerprints showed overlap with the supplementary motor area (SMA), bilateral supramarginal gyrus (PFop). The vPCC connectivity fingerprints showed overlap with the mid orbital gyrus (FP2), left middle frontal gyrus (Area 8b), bilateral prefrontal thalamus, left angular gyrus (PGp), and left inferior parietal lobule (hIP3). The RSC connectivity fingerprints only overlapped for the seed region.

**Supplemental Figure 1:** Histograms showing the Head Movement, Age, and FIQ distributions.

**
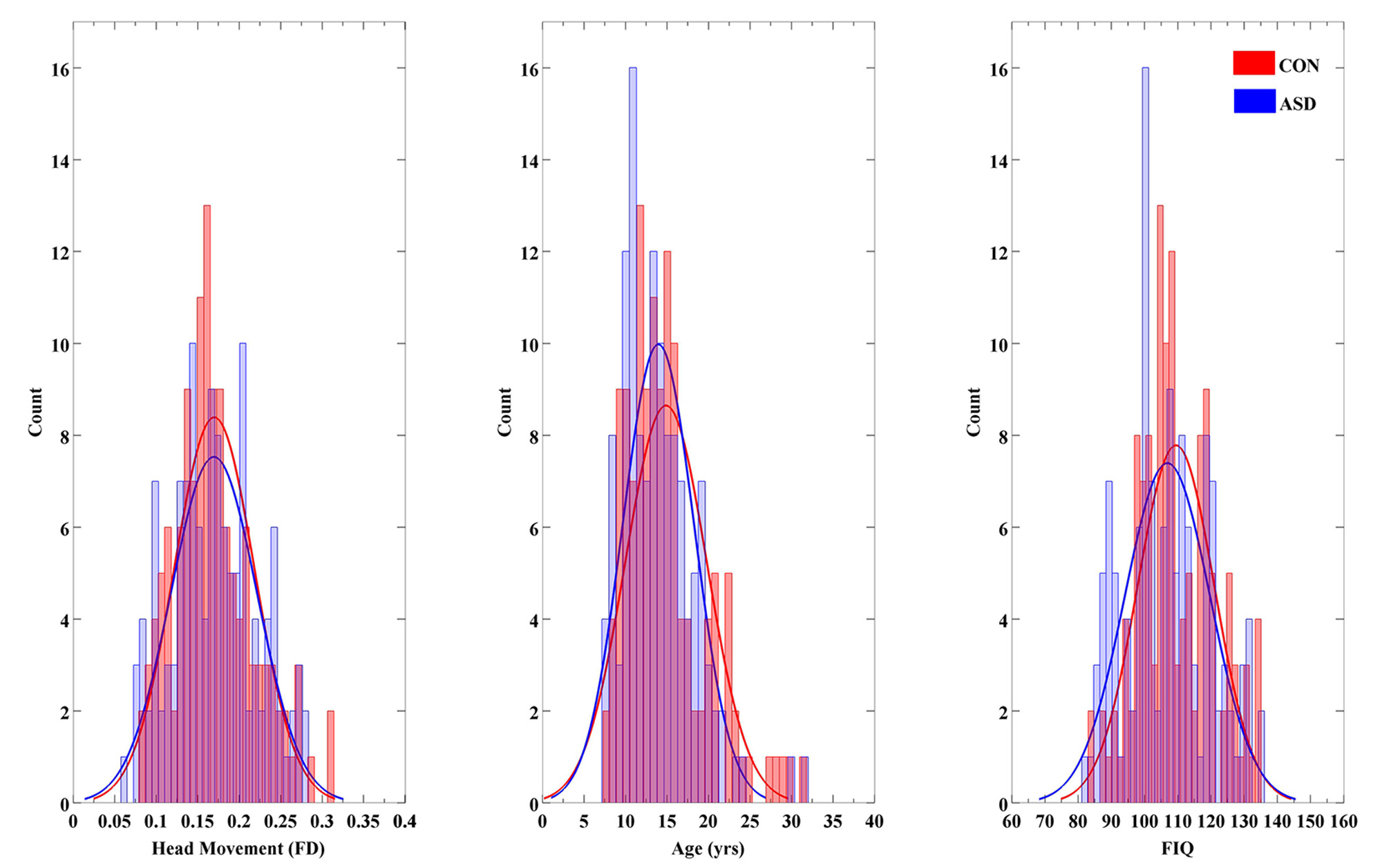
**

**Supplemental Figure 2:** a) MNI template, b) custom template made using DARTEL, c) MNI grey matter mask (white) with grey matter mask from custom template overlaid (red). In line with studies of brain maturation [Casey et al., 2008] the frontal lobe was much smaller in this sample than the MNI template would suggest.


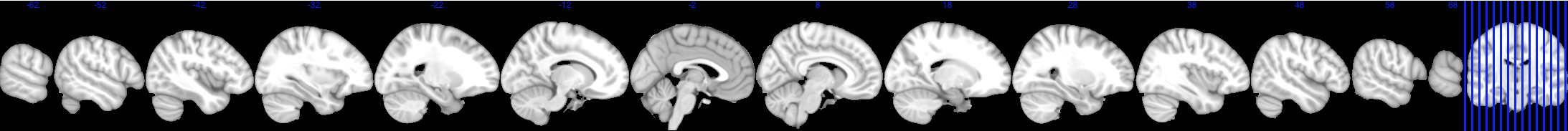

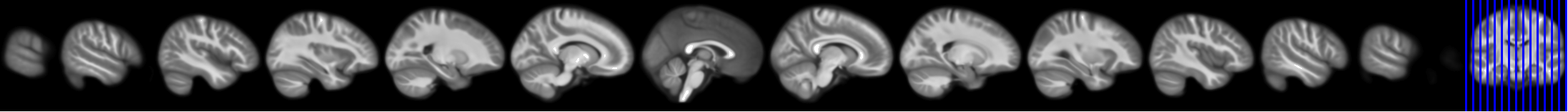

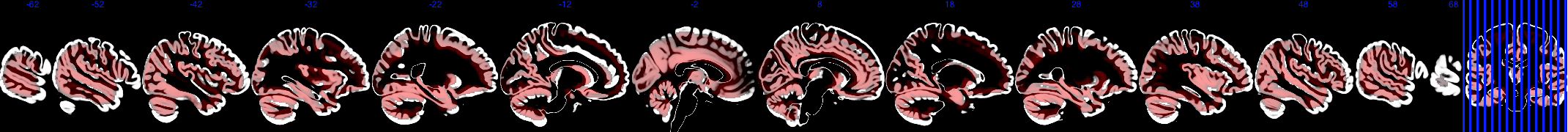

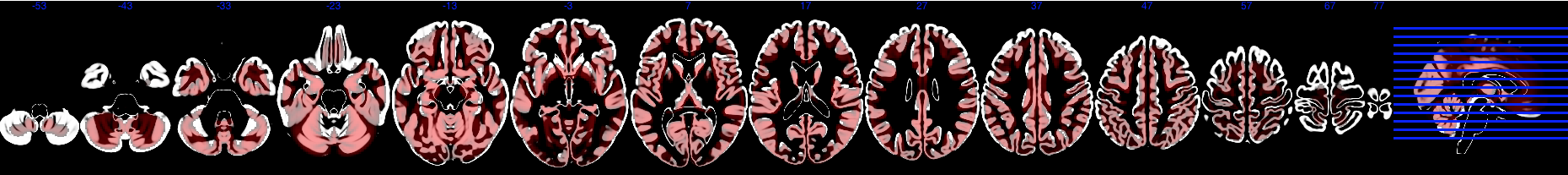


**Supplemental figure 3:** Two cluster solution showing the posterior cingulate (green) and anterior cingulate (magenta).

**
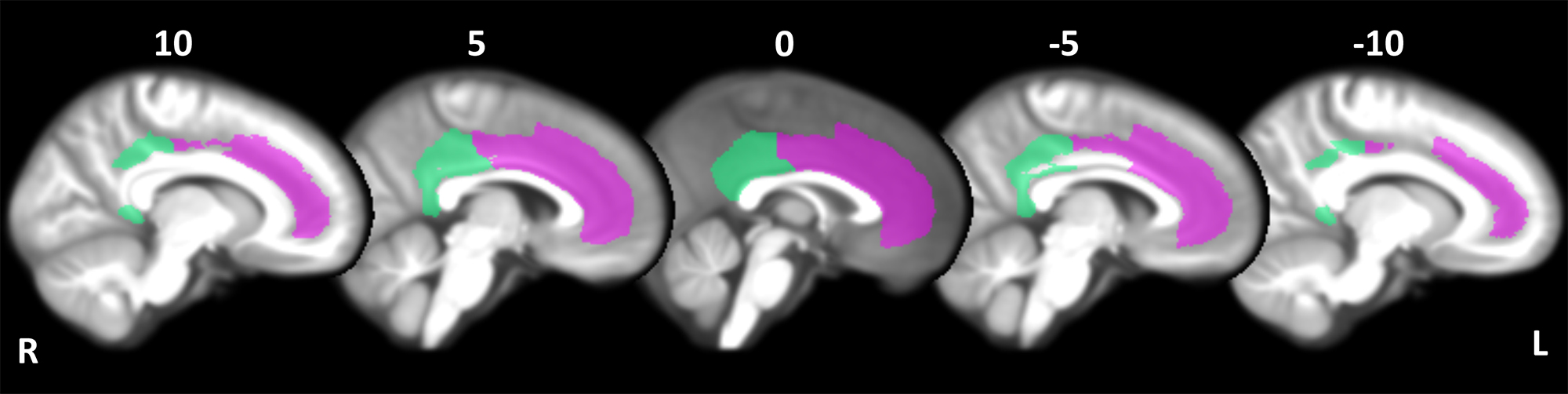
**

**Supplemental figure 4: MACM paradigm class profiles:** Bar plots show Bayesian probabilities for paradigm class associated with each cluster. Here we show the reverse inference, i.e. the likelihood of a paradigm class given the location of the activation cluster. Behavioural domains are contrasted with adjacent clusters. All paradigm class information has been corrected for multiple comparisons (FDR, P<0.05). A) ACC compared to aMCC. B) aMCC compared to pMCC. C) pMCC compared to dPCC. D) dPCC compared to vPCC. The colour of each bar corresponds to the colour code established in figure 2.

**
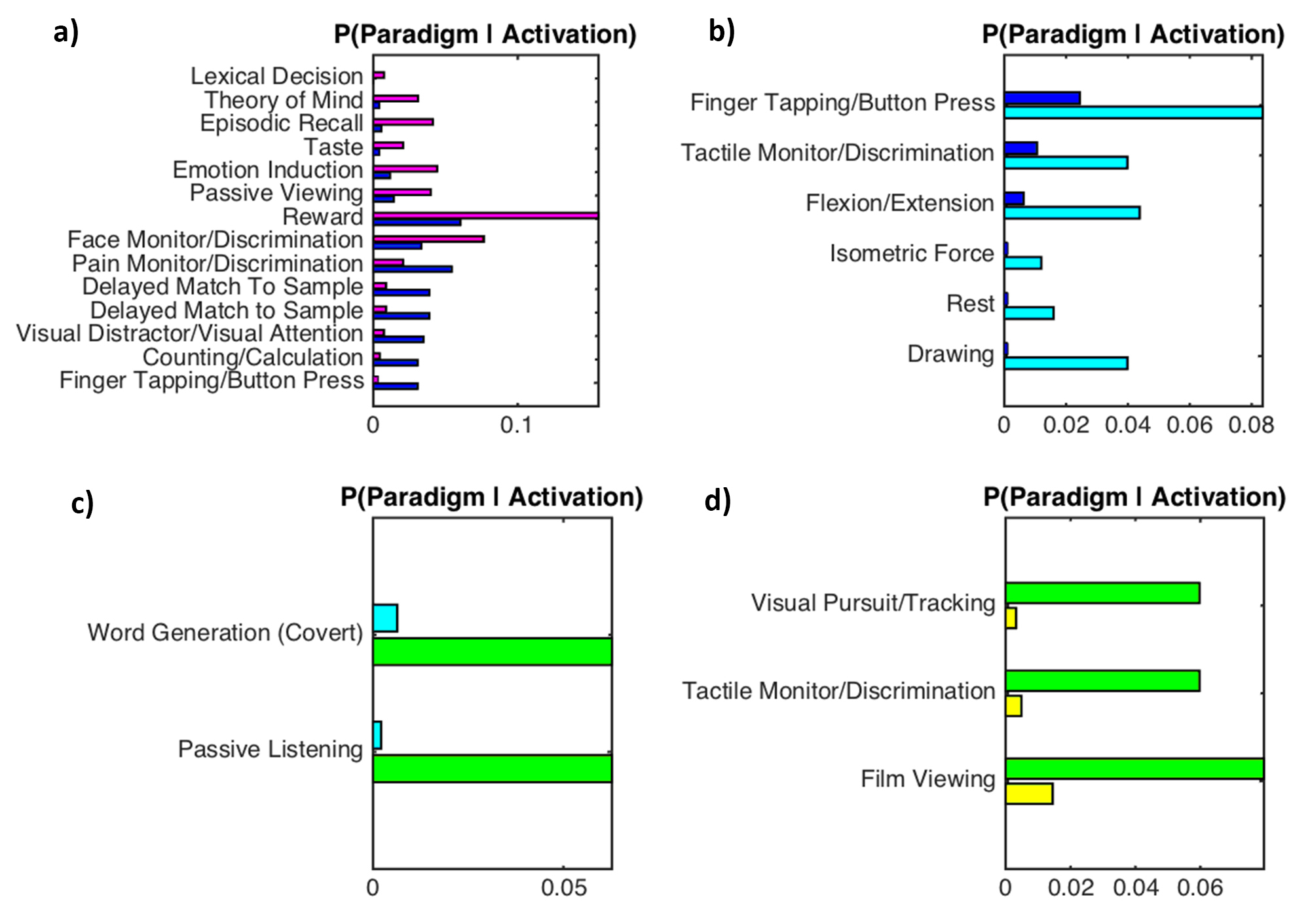
**

**Supplemental figure 5:** vPCC probabilty map partitioned into 24 2*2*2 voxel cubes (equivalent of a 4mm radius sphere). Average network strength (Z Score) for each of the 24 seeds is plotted below. Blue shows the seeds in sagital slice X=5, red for X = -2, green for X = -8. Within each sagittal slice the numbering goes from ventral to dorsal, then one seed anterior. Asterisks indicate a signficant group difference (p<0.05, uncorrected).

**
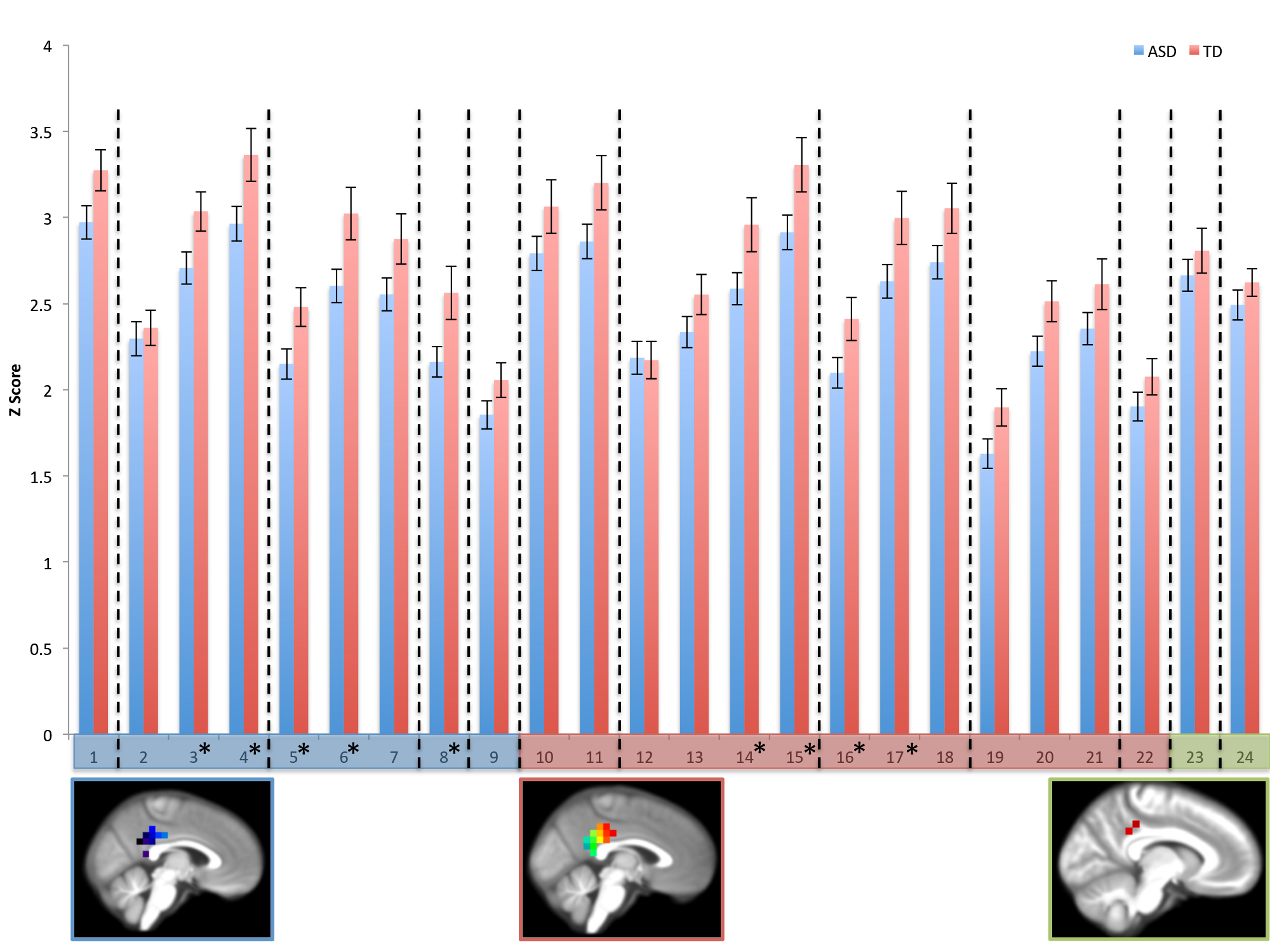
**

**Supplemental**  **Figure 6: Grey matter (GM) differences between ASD and TD.** A) GM differences overlaid on an inflated brain. The coloured lines highlight the boundaries of resting state networks established by Yeo et al (2011). B) bar plot showing the distribution of **voxels showing a significant group difference in GM (i.e. voxels illustrated in a)** across the 7 networks C) bar plot showing the percentage of the RSN network overlapping with the **group difference GM voxels (illustrated in a)**. In all cases the colours indicate the following networks: dark purple outlines the visual network, blue outlines the somatomotor network, green outlines the dorsal attention network, violet outlines the ventral attention network, cream outlines the limbic network, orange outlines the frontoparietal network (FPN), and red outlines the DMN. This same colour scheme was used by Yeo et al (2011).


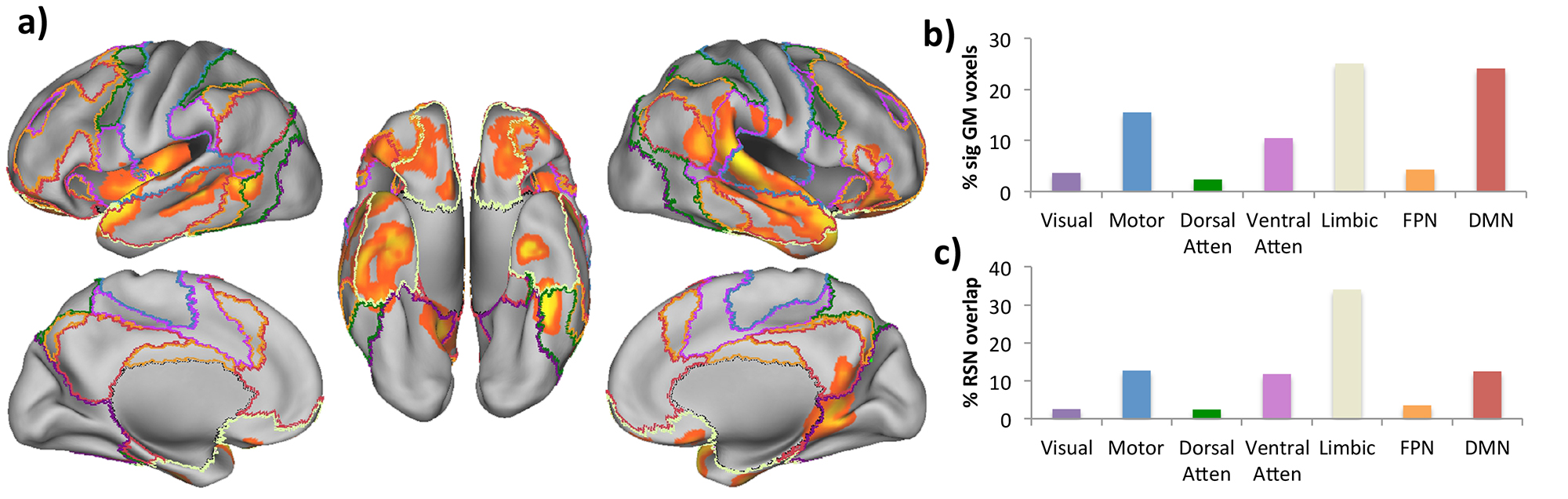


**Supplemental figure 7:** Blue regions show a negative relationship (increased connectivity with decreased age) between the ACC and age (p<0.05, TFCE FWE corrected). **
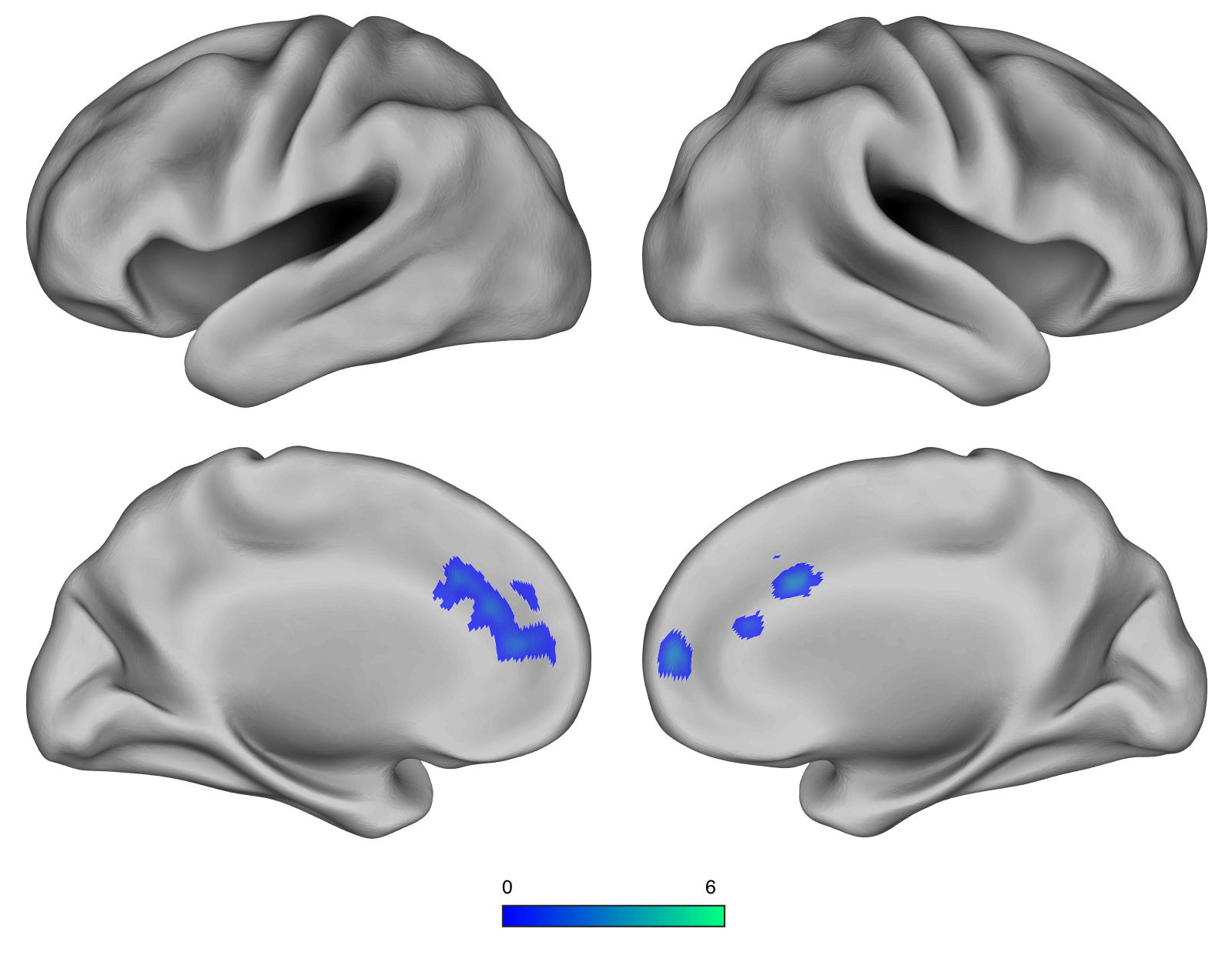
**

**Supplemental figure 8:**  Red regions show a positive relationship (increased connectivity with increased age) between the pMCC and age (p<0.05, TFCE FWE corrected). **
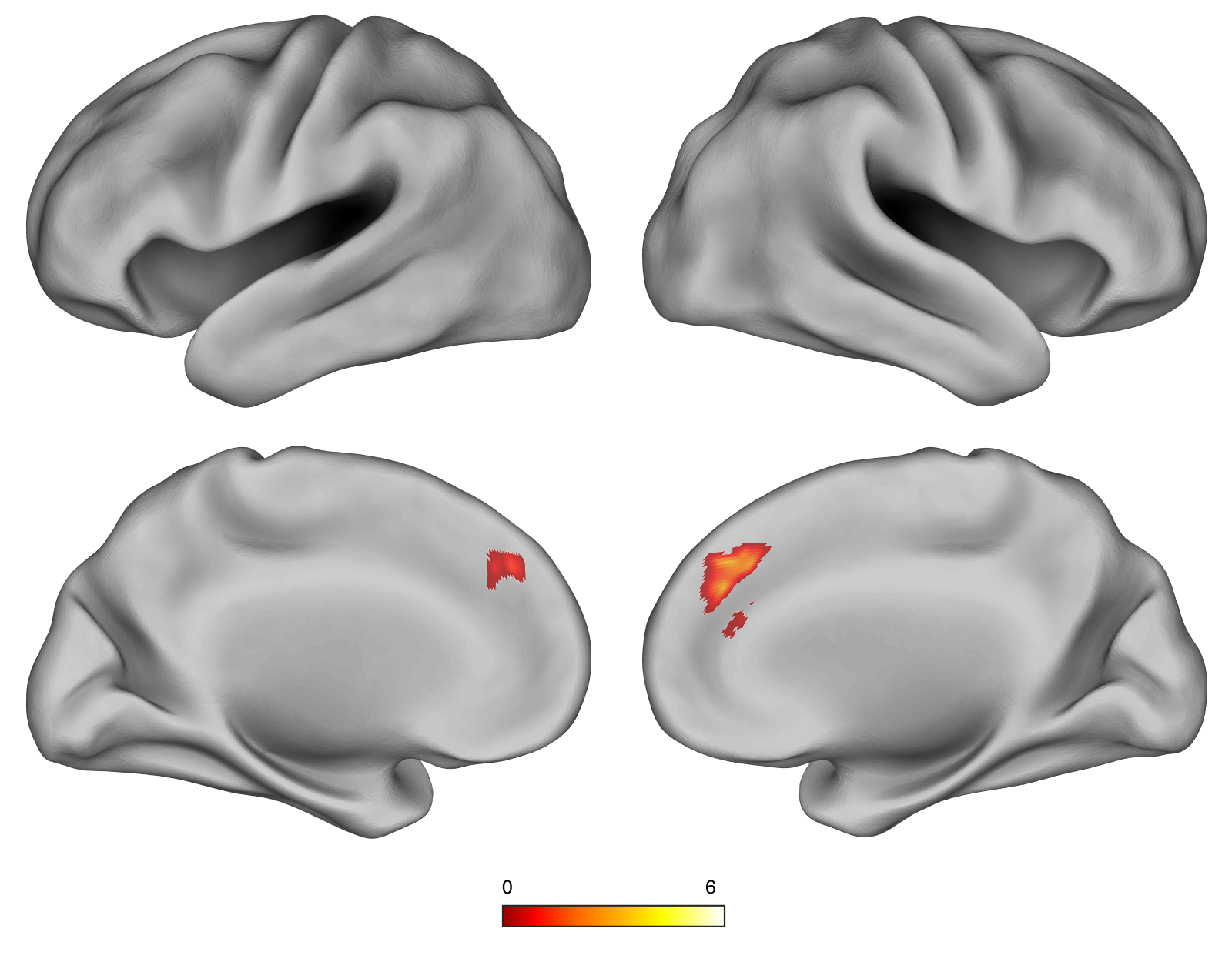
**

**Supplemental figure 9:** Red regions show a positive relationship (increased connectivity with increased age) between the dPCC and age. Blue regions show a negative relationship (increased connectivity with decreased age) between the dPCC and age (p<0.05, TFCE FWE corrected).

**
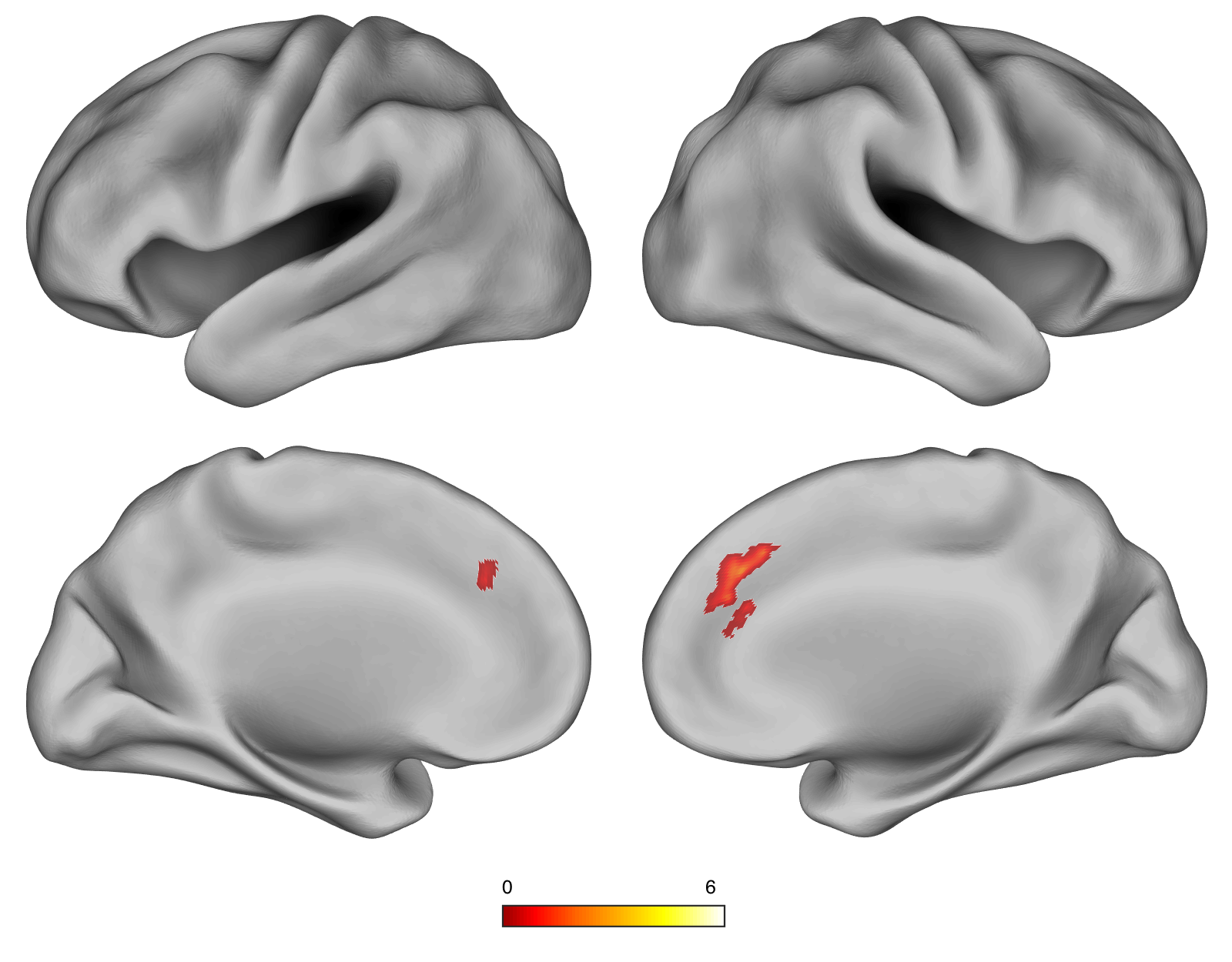
**

**
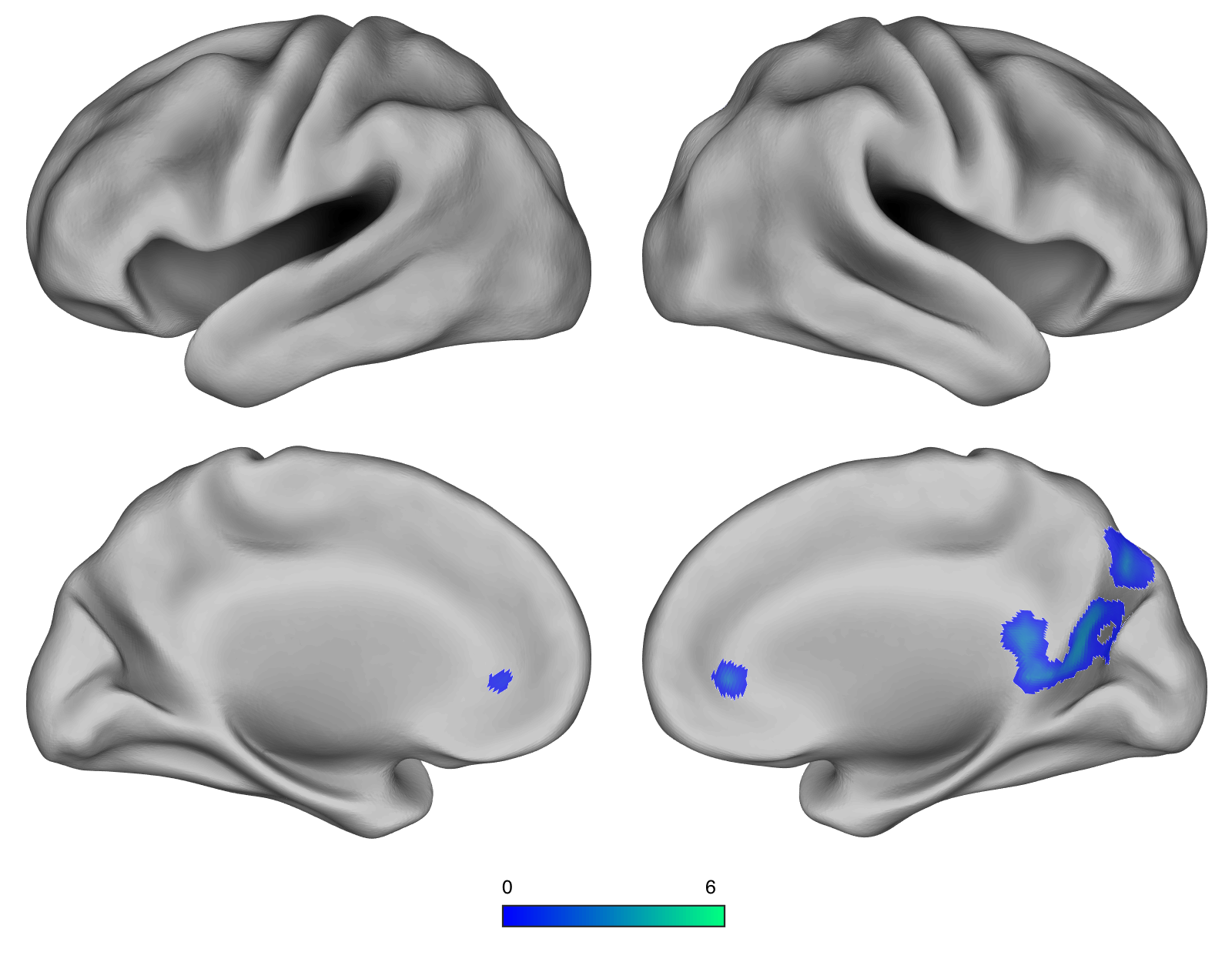
**

**Supplemental figure 10:** Red regions show a positive relationship (increased connectivity with increased age) between the vPCC and age (p<0.05, TFCE FWE corrected). **
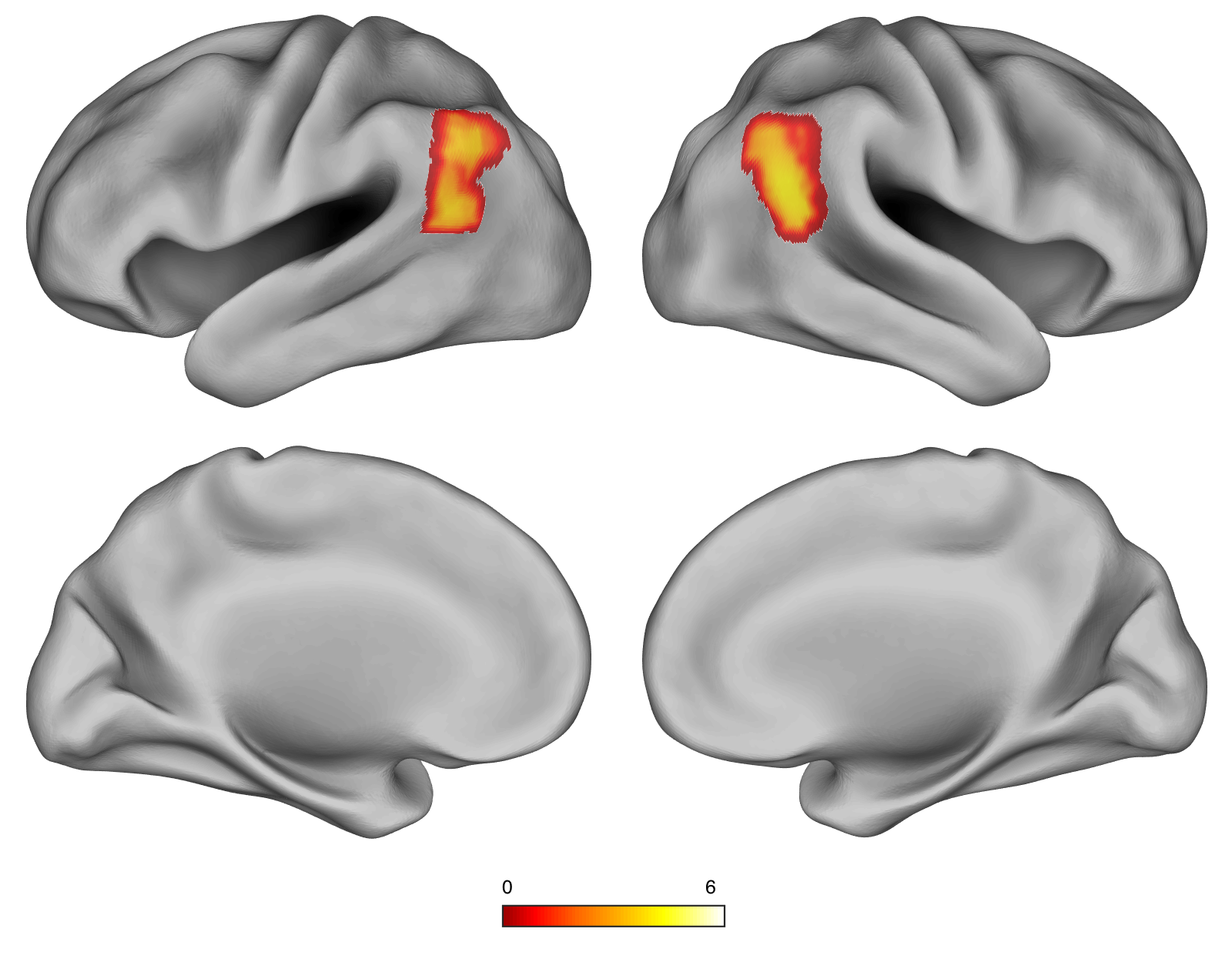
**

**Supplemental figure 11:** Blue regions show a negative relationship (increased connectivity with decreased age) between the RSC and age (p<0.05, TFCE FWE corrected). Red regions show a positive relationship (increased connectivity with increased GM) between the RSC and GM (p<0.05, TFCE FWE corrected).

**
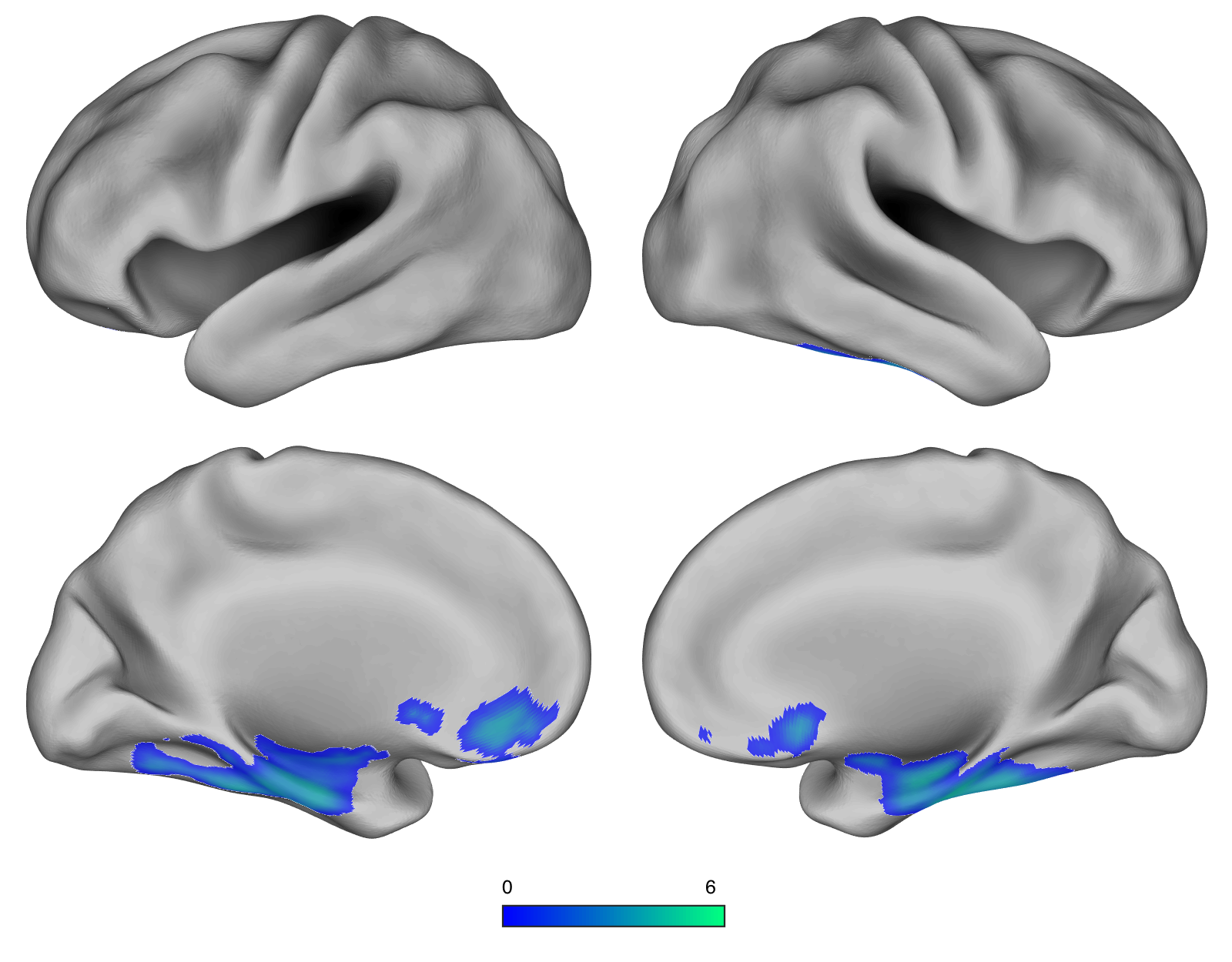
**

**
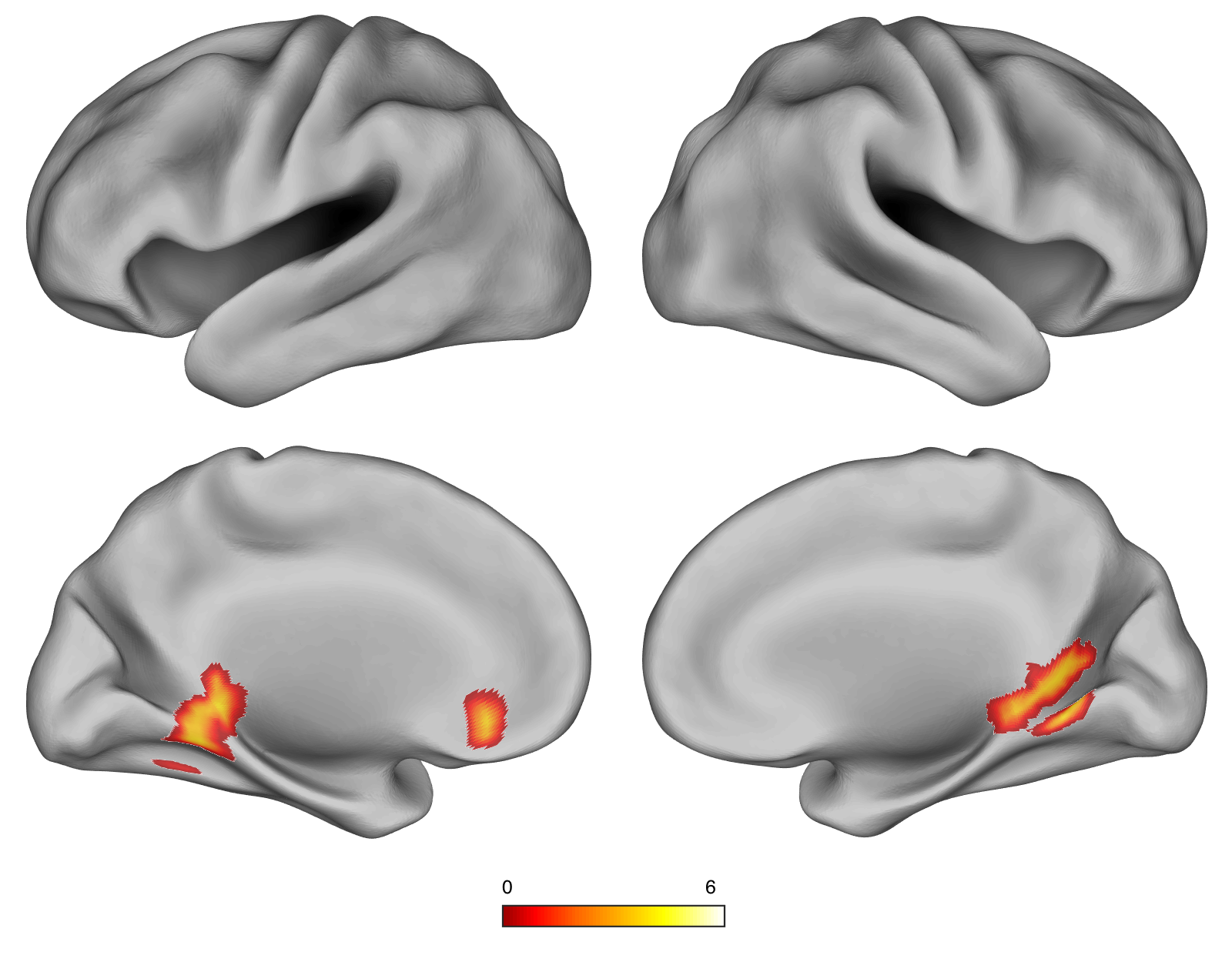
**

**Supplemental figure 12:** Anterior (blue) and posterior (cyan) MCC probability maps. The white dashed line shows that the separation of the aMCC and pMCC based on the VAC line.

**
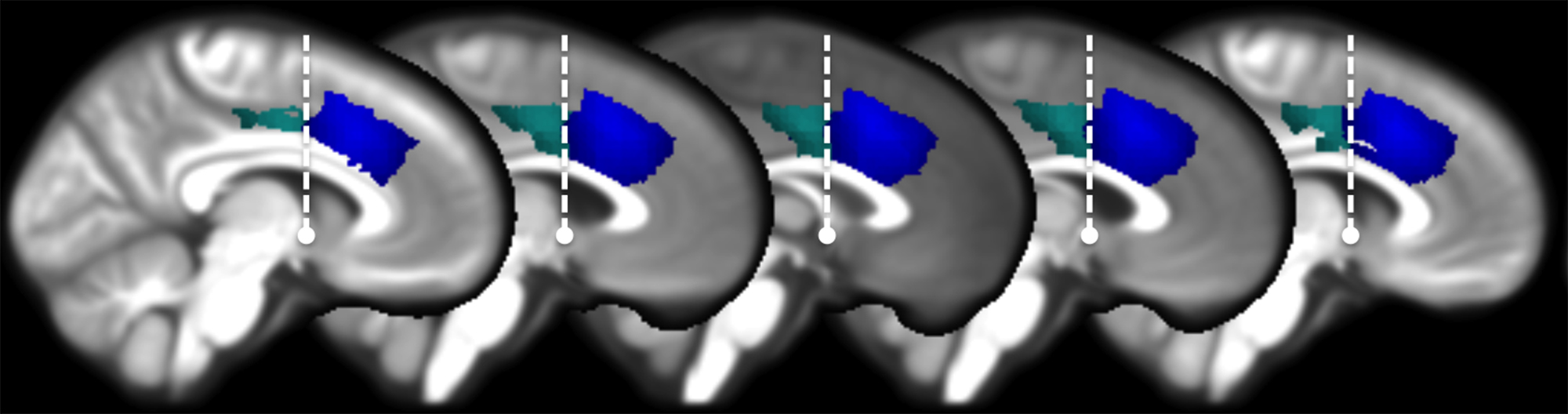
**

# References

Beckmann M, Johansen-Berg H, Rushworth MFS (2009): Connectivity-based parcellation of human cingulate cortex and its relation to functional specialization. Journal of Neuroscience 29:1175–1190.

Casey BJ, JONES RM, HARE TA (2008): The Adolescent Brain. Annals of the New York Academy of Sciences 1124:111–126.

Hoffstaedter F, Grefkes C, Caspers S, Roski C, Palomero-Gallagher N, Laird AR, Fox PT, Eickhoff SB (2013): The role of anterior midcingulate cortex in cognitive motor control. Hum Brain Mapp:n/a–n/a. http://onlinelibrary.wiley.com/doi/10.1002/hbm.22363/full.

Johansen-Berg H, Behrens T, Robson MD, Drobnjak I, Rushworth M, Brady JM, Smith SM, Higham DJ, Matthews PM (2004): Changes in connectivity profiles define functionally distinct regions in human medial frontal cortex. Proc Natl Acad Sci USA 101:13335–13340.

Picard N, Strick PL (2001): Imaging the premotor areas. Current Opinion in Neurobiology 11:663–672.

Yeo BTT, Krienen FM, Sepulcre J, Sabuncu MR, Lashkari D, Hollinshead M, Roffman JL, Smoller JW, Zöllei L, Polimeni JR (2011): The organization of the human cerebral cortex estimated by intrinsic functional connectivity. Journal of Neurophysiology 106:1125–1165.
